# Water Droplet Dynamics on a Hydrophobic Surface in Relation to the Self-Cleaning of Environmental Dust

**DOI:** 10.1038/s41598-018-21370-5

**Published:** 2018-02-14

**Authors:** Bekir Sami Yilbas, Ghassan Hassan, Abdullah Al-Sharafi, Haider Ali, Nasser Al-Aqeeli, Abdelsalam Al-Sarkhi

**Affiliations:** 1 Department of Mechanical Engineering, King Fahd University of Petroleum and Minerals (KFUPM), Dhahran, 31261 Saudi Arabia; 2 Center of Research Excellence in Renewable Energy (CoRE-RE), King Fahd University of Petroleum and Minerals (KFUPM), Dhahran, 31261 Saudi Arabia

## Abstract

The dynamic motion of a water droplet on an inclined hydrophobic surface is analyzed with and without environmental dust particles on the surface. Solution crystallization of a polycarbonate surface is carried out to generate a hydrophobic surface with hierarchical texture composed of micro/nanosize spheroids and fibrils. Functionalized nanosize silica particles are deposited on the textured surface to reduce contact angle hysteresis. Environmental dust particles are collected and characterized using analytical tools prior to the experiments. The droplet motion on the hydrophobic surface is assessed using high-speed camera data, and then, the motion characteristics are compared with the corresponding analytical results. The influence of dust particles on the water droplet motion and the amount of dust particles picked up from the hydrophobic surface by the moving droplet is evaluated experimentally. A 40 μL droplet was observed to roll on the hydrophobic surface with and without dust particles, and the droplet slip velocity was lower than the rotational velocity. The rolling droplet removes almost all dust particles from the surface, and the mechanism for the removal of dust particles from the surface was determined to be water cloaking of the dust particles.

## Introduction

Climate change increases the frequency of regular dust storms around the globe, particularly in the Middle East and the Sahara region. The performance of a solar energy harvesting device is highly dependent on the amount of incident solar energy reaching the active surface of the device. The settling of dust on these surfaces degrade the device performance in terms of efficiency and power output^1^, and additional effort is required to remove the dust. Several methods have been proposed to remove dust from surfaces, including sonic and mechanical excitation of the dust particles^2^, mechanical brushing^3^, air jet blowing^4^, and water cleaning^5^, among others. Most of these methods require sophisticated devices or external effort, such as excess energy, to operate. The scarcity of clean water limits practical applications of water jet and water film cleaning of surfaces, and hence, water consumption should be minimized during cleaning. On the other hand, adopting self-cleaning surfaces can minimize external energy usage in energy harvesting devices and provide an effective cleaning process for removing dust particles. In general, the self-cleaning process utilizes surfaces with hydrophobic characteristics, where the low free energy of the surface and reduced particle contact area at the surface, due to air gaps within the surface texture, allow only weak adhesion of particles on the surface. In addition, the low contact angle hysteresis of hydrophobic surfaces enables water droplet rolling on an inclined hydrophobic surface. This, in turn, facilitates the removal of weakly adhered dust particles from the hydrophobic surface by rolling water droplets. However, many factors affect droplet rolling and self-cleaning on surfaces, including the size, shape, elemental composition, and density of the dust particles; the roughness, contact angle hysteresis and inclination angle of the surface; and the droplet volume. The acceleration of a water droplet on an inclined hydrophobic surface and the droplet residence time for picking up the dust particles are important for achieving clean surfaces free of dust residue. Consequently, investigation of the acceleration of water droplets on inclined hydrophobic surfaces for the removal of dust particles from the surface becomes essential.

Many research studies have examined surface hydrophobicity and related characteristics. The surface hydrophobicity mainly depends on the interfacial energies of the solid and liquid, the surface texture, and the Laplace pressure of the liquid droplet^6,7^. The surface hydrophobicity of a substrate can be considerably improved by chemical modification and by increasing the surface roughness^8^. Several techniques have been proposed and used to hydrophobized surfaces^9–13^, but some of these techniques are associated with multi-step processes involving harsh conditions or specialized reagents. In addition, oil impregnation can be introduced on a textured surface to improve the optical transmittance while achieving the self-cleaning effect on the surface^14^. Because self-cleaning requires the surface to be hydrophobic, most hydrophobicity studies are focused on achieving self-cleaning effects on the surface^15–22^.

Several studies have investigated water droplet mobility, rolling and impact on hydrophobic surfaces^23–26^. Some studies considered droplet mobility through thermal excitation of the liquid droplet utilizing the thermocapillary effect^27^. In this case, rotating circulation cells were formed inside the droplet due to the combination of the Marangoni and buoyancy currents. This, in turn, gave rise to the droplet fluid inertia force, which became greater than the adhesion and fluid shear forces during the thermal excitation period^27^. For textured isothermal hydrophobic surfaces, both the static contact angle and rolling angle increased with an increase in the micropillar height. When the micropillar spacing was increased, the rolling angle decreased; however, the change in the static contact angle was irregular. On superhydrophobic surfaces, the spreading coefficient of the droplet was affected by both the static contact angle and the rolling angle, and the rebounding coefficient of the droplet was highly associated with the rolling angle, where the bigger the inclination angle of the surface, the smaller the rebounding coefficient of the droplet^28^. Newer models are suggested to overcome this shortcoming of current models by incorporating the adhesion energy of a droplet using the relationship between the solid and liquid contact areas^29^. Although hydrophobic surfaces show significant asymmetry in the advancing-to-receding profiles of droplets, superhydrophobic surfaces demonstrate advancing-to-receding profiles with different behavior^30^. This, in turn, results in larger force requirements for droplet rolling than that on superhydrophobic surfaces. In addition, the lateral retention (adhesion) forces developed on hydrophobic surfaces are much higher than those formed on superhydrophobic surfaces^30^. Consequently, the inclination angle for droplet rolling to achieve self-cleaning is lower for superhydrophobic surfaces than for hydrophobic surfaces. However, sliding can occur during rolling, which influences the dynamic characteristics of rolling droplets^31^. Asymmetrically patterned surfaces can favor preferential droplet transport through sliding^32^. In this case, the unbalanced capillary force developed at the contact line becomes critical for achieving a preferable direction of liquid motion. However, droplet sliding on an asymmetrically structured surface can result in different migration velocities, which depend on the direction of the structure with respect to droplet movement^32^. Therefore, applications of droplet sliding for self-cleaning purposes require the proper design of textural features that enable control of the sliding direction.

Environmental dusts have various shapes and sizes and can scatter and absorb incident solar radiation while reducing the amount of solar radiation that reaches the surface. The scattering and absorption of incident radiation becomes detrimental to the solar harvesting device as the thickness of the dust layer increases on the surface over time. Therefore, the removal of dust from the surface becomes essential to sustain the efficient of operation of solar harvesting devices. On the other hand, the dynamic motion of a water droplet remains critical for removing dust particles from hydrophobic surfaces. Although many research studies have examined water droplet rolling on hydrophobic surfaces^23–25^ and environmental dust characteristics^33–35^, their main focus was the droplet dynamics during rolling and the assessment of the environmental dust characteristics. Dust removal from inclined hydrophobic surfaces by water droplet rolling is often left for future study. In addition, droplet addition to hydrophobic surfaces and droplet impingement on the self-cleaning characteristics of surfaces have been studied previously^36^. The rolling dynamics of the droplet and the mechanism of dust removal from the hydrophobic surface, which involves cloaking of the dust particles by water, were left for future investigations. The state of water droplet and its dynamics on hydrophobic surfaces and formulation of droplet motion on the inclined surfaces were presented previously^37–42^; in which case, the influence of dust particles, located on the droplet path, on the dynamic characteristics of droplet is not the focus in the previous studies. Dust particles can modify the droplet dynamics on the surface while influencing the performance of the self-cleaning capacity of droplet on the hydrophobic surface. Consequently, in the present study, dust removal from an inclined hydrophobic surface is analyzed, and the droplet dynamics influencing the dust removal process are examined. The cloaking and wetting state of the dust particles by the water droplet are investigated in relation to the dust removal process. Solution crystallization of a polycarbonate surface is carried out to create a hierarchical, textured surface composed of micro/nanosize spheroids and fibrils. Synthesized silica nanoparticles are deposited onto the textured polycarbonate surface to create hydrophobic characteristics, reduce the contact angle hysteresis and generate the lotus effect on the surface. The environmental dust particles collected locally are characterized using analytical tools. The optical performance of the self-cleaned surfaces is assessed through UV-visible transmittance tests.

## Experimental

### Solution Crystallization of Polycarbonate Surface

A polycarbonate sheet with dimensions of 30 mm × 200 mm × 3 mm (width × length × thickness) was used as the base. Polycarbonate was derived from p-hydroxyphenyl, which was initially solution crystallized using acetone to create hierarchical textures on the surface. Polycarbonate wafers were ultrasonically cleaned prior to immersion in acetone for 2 minutes. Several tests were carried out to select the appropriate concertation of acetone and the appropriate immersion duration for crystallization of the polycarbonate surface. An acetone concentration of 60% (by volume in water) and immersion duration of 3 minutes were selected in line with a previous study^43^. This arrangement resulted in hierarchical crystal structures with hydrophobic characteristics on the surface.

### Functionalized Silica Particles Deposition

To generate the lotus effect at the surface to reduce the contact angle hysteresis, functionalized nanosize silica particles were deposited on the solution-crystallized polycarbonate surface. The silica nanoparticles were synthesized using a procedure similar to that reported in a previous study^44^. In this case, tetraethyl orthosilicate (TEOS), isobutyltrimethoxysilane (OTES), ethanol, and ammonium hydroxide were used in the synthesis. Prior to the deposition of functionalized silica particles, the crystallized polycarbonate surface was washed with piranha solution followed by distilled water to further clean the surface. Solvent casting was applied to deposit the solution onto a glass surface. After all of the solvent was evaporated by vacuum drying, the resulting surface was characterized. The dynamic contact angle of the resulting surface was measured in line with a previous study^45^, and a contact angle on the order of 158° with contact angle hysteresis on the order of 2° to 3° was measured. Consequently, the deposition of functionalized silica particles on the crystallized polycarbonate surface resulted in a superhydrophobic surface with significantly reduced contact angle hysteresis.

### Characterization Tools

The following analytical tools were used for characterization of the surface. A JEOL 6460 scanning electron microscope with a tungsten filament, beam voltage of 0.4–40 kV, resolution of 10 nm, and magnification of 10× to 300 000× was used. Atomic force microscopy/scanning probe microscopy (AFM/SPM) in contact mode was incorporated to analyze the surface texture. The tip was made of silicon nitride (*r* = 20–60 nm) with a manufacturer-specified force constant, k, of 0.12 N/m. The water droplet contact angle for functionalized silica particles deposited on an oil-impregnated surface was measured. During the measurements, the droplet volume was controlled by an automatic dispensing system with a volume step resolution of 0.1 μl. Still images were captured, and contact angle measurements were performed one second after deposition of the water droplet on the surface, in line with a previous study^45^.

### Environmental Dust Particles Collection and Analysis

Dust particles were collected over a period of 12 months from the energy laboratory of King Fahd University of Petroleum and Minerals, which is located close to the city of Dammam in Saudi Arabia. Dust particles accumulated on the surface of the protective glass of photovoltaic panels were removed by soft brushes and stored in an airtight container. The collected dust particles were first analyzed in terms of weight, size, shape, and elemental composition using the abovementioned analytical tools. The findings revealed that the dust particles collected over a one-week period within 12 months had similar characteristics in terms of elemental composition, size distribution, and shape. The amount of dust particles accumulated on the surface of the photovoltaic protective layer within one month was on the order of 20 g/m^2^; however, this number varied within 16% (by weight) over six months, which was attributed to the wind speed and direction. Although the wind speed and direction changed over time, the average wind speed was approximately 4 m/s over the year. The dust particles were characterized using SEM (JEOL 6460) and energy dispersive spectroscopy (EDS), which consisted of an INCA Mics microscope image capture system, including an INCA X-stream, X-ray acquisition, and detector control unit. X-ray photoelectron spectrometer (XPS) was performed incorporating ESCALAB 220 XL spectrometer. A monochromatic Al K_α_ X-ray source (1486.6 eV) was operated in the constant analyzer energy mode (CAE = 100 eV for survey spectra and CAE = 40 eV for high resolution spectra). X-ray diffraction (XRD, Model: D8 Advanced diffractometer, Manufacture: Bruker, USA) analysis was performed with CuKα radiation at typical settings of 40 kV and 30 mA. Water cloaking of the dust particles was monitored, and the cloaking velocity was measured using a high-speed camera (Model: SpeedSense 9040, Manufacture: Dantec Dynamic, Denmark). The optical transmittance of the surface after dust removal by the water droplet was measured using a UV spectrophotometer (Model: 67 Series, Manufacture: Jenway, UK), and the result was compared with that of the surface deposited with functionalized silica particles.

## Results and Discussion

The droplet transition dynamics on a hydrophobic surface are pertinent to environmental dust removal from the surface. Polycarbonate surfaces were solution crystallized to generate hierarchical micro/nanosize textures. To reduce the contact angle hysteresis, functionalized silica nanoparticles were deposited on the textured surface, which results in superhydrophobic surfaces with low contact angle hysteresis. Water droplet movement on the resulting inclined surface with and without environmental dust particles was analyzed in relation to self-cleaning applications. The dust particles were characterized using the abovementioned analytical tools prior to experiments.

### Characteristics of the Dust Particles

**Figure 1:**
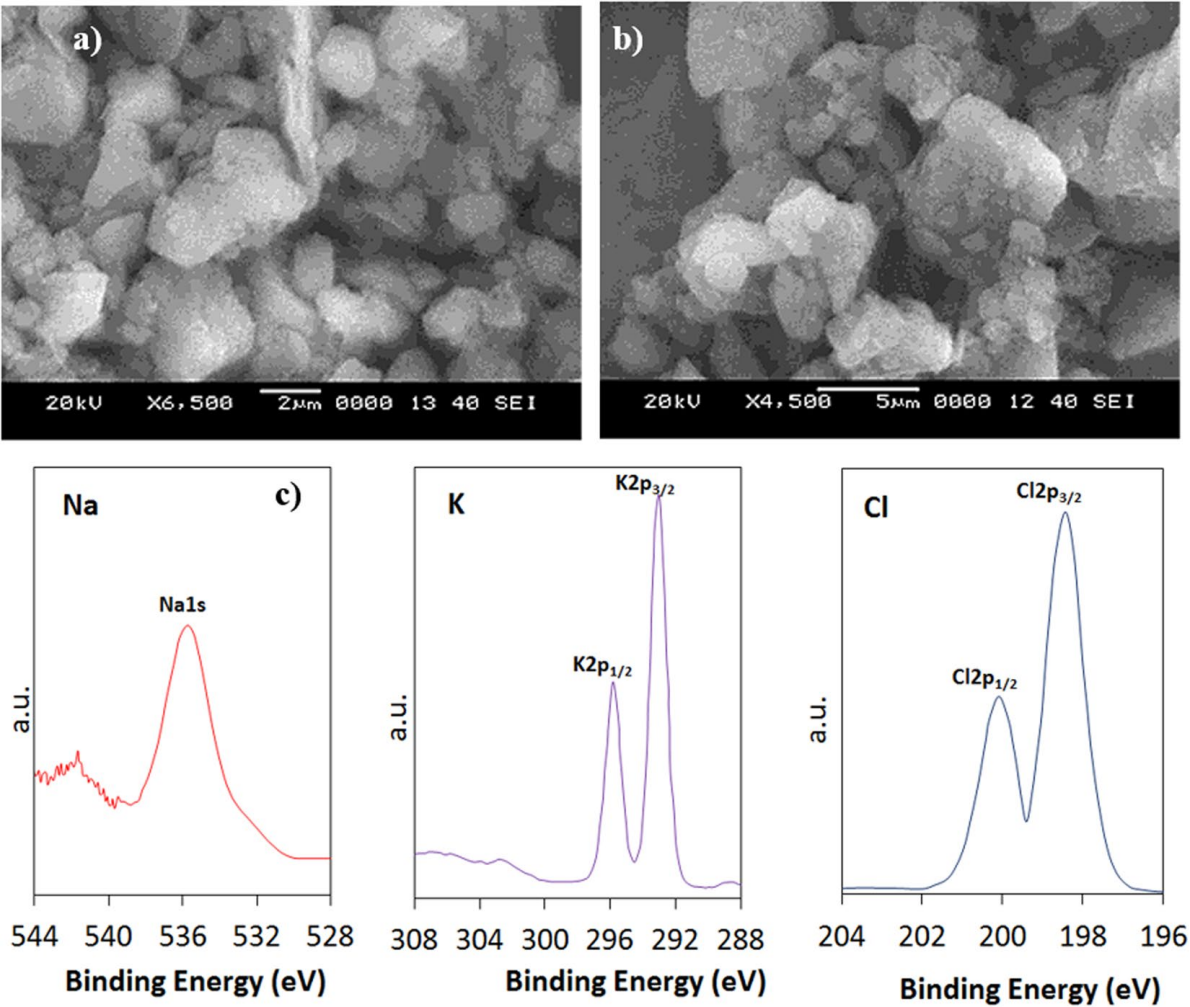
SEM micrographs of the dust particles: (**a**) particles with various shapes and sizes, (**b**) small dust particles attached to the surface of large particles, XPS peaks for binding energy of sodium, potassium, and chlorine.

**Figure 2:**
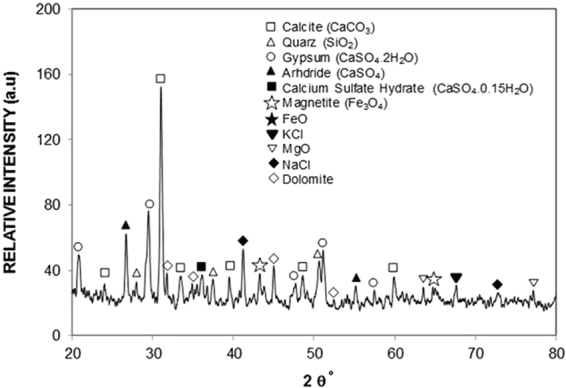
X-ray diffractogram of the dust particles.

Figure (1) shows SEM micrographs of the dust particles collected in the local area of Dammam, Saudi Arabia. The particles have various sizes and shapes (Fig. (1a)). The size of the dust particles varies within the range of 0.1 μm to 15 μm, and the average size of the dust particles is on the order of 1.2 μm. Some of the smaller dust particles, which are in the sub-micrometer range, attach to the surfaces of the larger dust particles (Fig. (1b)). The bright areas, which are typically observed for the smaller particles, are indicative of electron charging during SEM analysis. This indicates that the small particles are charged prior to SEM analysis. Therefore, the initial charges of the small particles create forces for attachment to the surfaces of the large particles. The geometric features of the dust particles can be characterized by the shape factor and the aspect ratio^37^. The shape factor is described by ${R}_{Shape}=\frac{{P}^{2}}{4\pi A}$, where *P* is the perimeter of the dust particle, and the aspect ratio is described by ${A}_{Aspect}=\frac{\pi {({L}_{proj})}^{2}}{4A}$, where *A* is the cross-sectional area and *L*_proj_ is the longest projection length of the dust particle. The shape factor represents the ratio of the major-to-minor axes of an ellipsoid that is best fit to the particle. The aspect ratio is associated with the approximate particle roundness and is inversely proportional to the particle circularity, which is associated with the complexity of the particle (i.e., a shape factor of unity corresponds to a perfect circle). The relationship between the particle size and the aspect ratio or the shape factor is complicated due to the irregular shapes and varying sizes of the dust particles. Nevertheless, an inverse relationship is observed between the particle size and the aspect ratio, whereas a direct relationship is observed between the particle size and the shape factor. Hence, the particle aspect ratio decreases as the shape factor increases, which is more pronounced for the large-size particles. The cross-sectional area of a typical dust particle 1.8 μm in size is on the order of 2.5 μm^2^, which results in a shape factor of 1.05. However, the cross-sectional area of a typical dust particle 15 μm in size is on the order of 175 μm^2^, and the corresponding shape factor is approximately 3.18. The shape factor of the small particles (<1.2 μm) approaches unity, while the median shape factor of the large particles (≥10 μm) approaches 3. The elemental composition of the dust particles is given in Table 1. The most common elements in the dust particles are Si, Ca, Mg, Na, K, S, O, and Fe, irrespective of the size and shape of the dust particles; moreover, the concentrations of Na, K, Ca, and O are found to increase, and chlorine is also present in the small particles (<1.2 μm). The changes in the elemental composition of the small particles (<1.2 μm) can be attributed to their prolonged residence time in the atmosphere, during which long periods of interaction occur between solar radiation and the small dust particles. Hence, the prolonged residence of small-size particles in the atmosphere allows for the attachment of ionic compounds in regions near the Gulf Sea. The concentration of chlorine varied among the different small dust particles, and the EDS data are not consistent with the molar ratio of NaCl, as given in Table 1. The chlorine concentration in the small dust particles suggests that the dust particles do not contain salt crystals but rather chlorine dissolved in compounds. Since the concentrations of K, Cl and, Na are low in the dust particles (Table 1), XPS is carried out determine the concentrations in the dust particles. Figure (1c) shows the XPS data for the binding energy of K, Cl, and Na. The potassium, sodium, and chlorine on the outermost layers of the dust particle is evident from XPS data and chlorine comprises of inorganic chloride; in which case, the Cl2p_3/2_ peak intensity occurs at 198.9 eV, which is in good agreement with the previous study^46,47^. XPS peak for potassium K2p_3/2_ occurs at 293.2 eV. The binding energy data for potasium K2p_3/2_ and Cl2p_3/2_ peaks demonstrates the presence of KCl in the dust. The concentration analysis via using CasaXPS indicates that the concentration ratio (after transforming in mass percentage) for K over Cl is in the order of 1.62, which is similar to that obtained from Table 1. Similar measurements are carried out for sodium and XPS binding data for Na1s peak occurs at 1072.8 eV (Fig. (1c)), which agrees with the previous data reported^47,48^. The concentration analysis via using CasaXPS reveals that the concentration ratio (after transforming in mass percentage) for Na over Cl is in the order of 2.67, which is similar to that given in Table 1. In order to observe the compounds in the dust particles, XRD is carried out. Figure (2) shows the X-ray diffractogram of the dust particles. Peaks corresponding to K, Na, Ca, S, Cl, and Fe are evident in the diffractogram. The iron peak coincides with the aluminum and silicon peaks, and the sodium and potassium peaks are likely associated with sea salt, as the dust particles are collected from a region near the Gulf Sea. The presence of sulfur may be related to the presence of calcium, such as anhydrite or gypsum components (CaSO_4_), in the dust particles. Iron is likely associated with clay-aggregated hematite (Fe_2_O_3_).

**Table 1:** Elemental composition of dust (wt.%) determined by energy dispersive spectroscopy (EDS).

	Si	Ca	Na	S	Mg	K	Fe	Cl	O
Size ≥ 1.2 μm	12.3	8.2	2.1	1.4	2.6	0.7	1.1	0.3	Balance
Size <1.2 μm	10.1	7.1	3.1	2.4	1.1	1.8	1.2	1.2	Balance
Dust Residues	12.5	8.1	2.1	1.3	2.4	0.9	1.0	0.4	Balance

**Figure 3:**
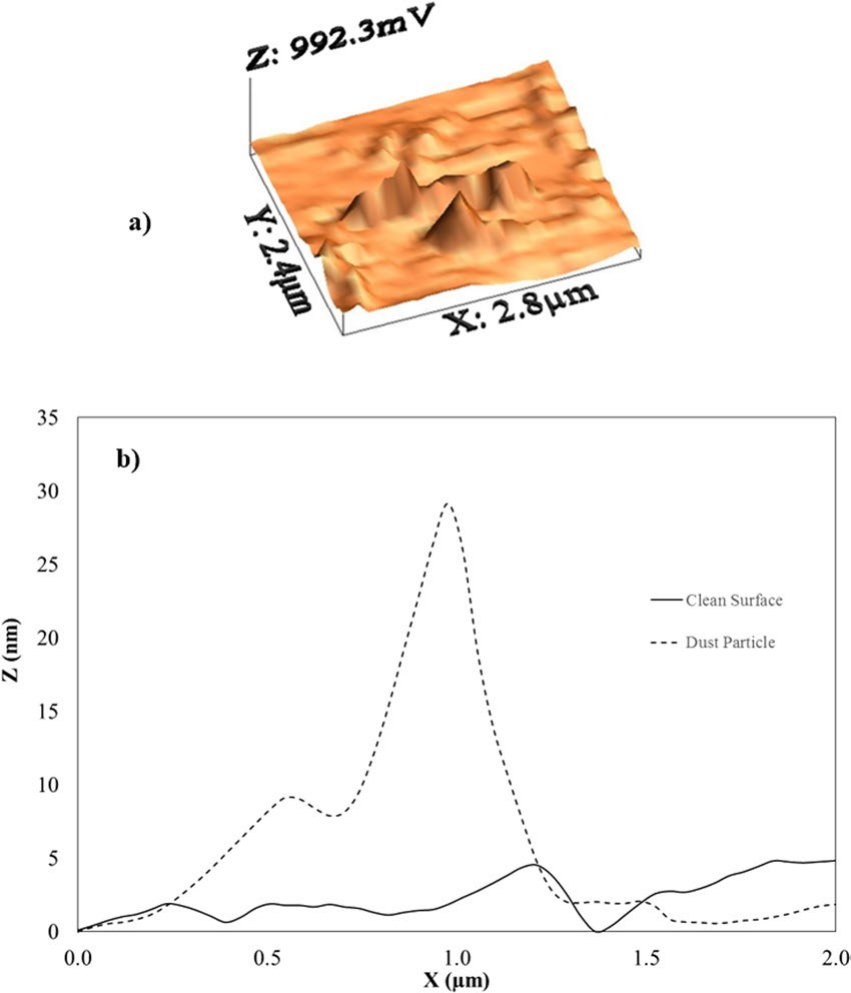
AFM data of the dust particles: (**a**) friction data for the dust particles and (**b**) retention force of the dust particle. The frictional force is also shown for the clean surface.

To assess the adhesion of dust particles to the hydrophobic surface, tangential force measurements for dust particle displacement on the hydrophobic surface were carried out by AFM^14^. When small-size dust particles attach on the surface of large-size particles, they agglomerate to form small dust clusters. The small-size dust particles have a charge field due to the prolonged duration of exposure to the atmosphere near the Gulf Sea, and the charges enhance the adhesion of these particles to the hydrophobic surface. The dust particles possess alkaline (Na, K) and alkaline earth (Ca) compounds (Table 1). These compounds contribute to ionic bonding at the surface under the influence of humidity while enhancing adhesion between the dust particles and the hydrophobic surface. Therefore, the presence of ionic bonding and electrostatic charge forces between the dust particles and the hydrophobic surface modifies the retention force. AFM was used to measure the retention force due to adhesion of a dust particle on the hydrophobic surface, where the AFM cantilever tip is proportional to the slope of the deflection of the tip when the tip is in contact with the surface. In this case, from the deflection relation, the retention force can be written as $\,F=k\sigma {\rm{\Delta }}V$^14^, where *k* is the spring constant of the cantilever tip (N/m), *σ* is the slope of the displacement over the recorded probe voltage (*Δz*/*ΔV*, m/V), and *ΔV* is the voltage recorded during surface scanning by the AFM tip in the contact mode. In the measurements, the following values were adopted: *k* = 0.12 N/m and Δz/ΔV = 1.481×10^−6^ m/V. Figure (3a) shows the AFM friction data of a dust particle on the solution-crystallized surface and that with deposited functionalized silica particles, while Fig. (3b) shows the retention force data in terms of mV. The maximum value of the retention force obtained from AFM analysis is on the order of 30 nN for a single dust particle with an average size of 1.2 μm; however, the retention force for a single dust particle with an average size of 5.2 μm on the hydrophobic surface is on the order of 65 nN.

### Surface Texture and Hydrophobic Characteristics

**Figure 4:**
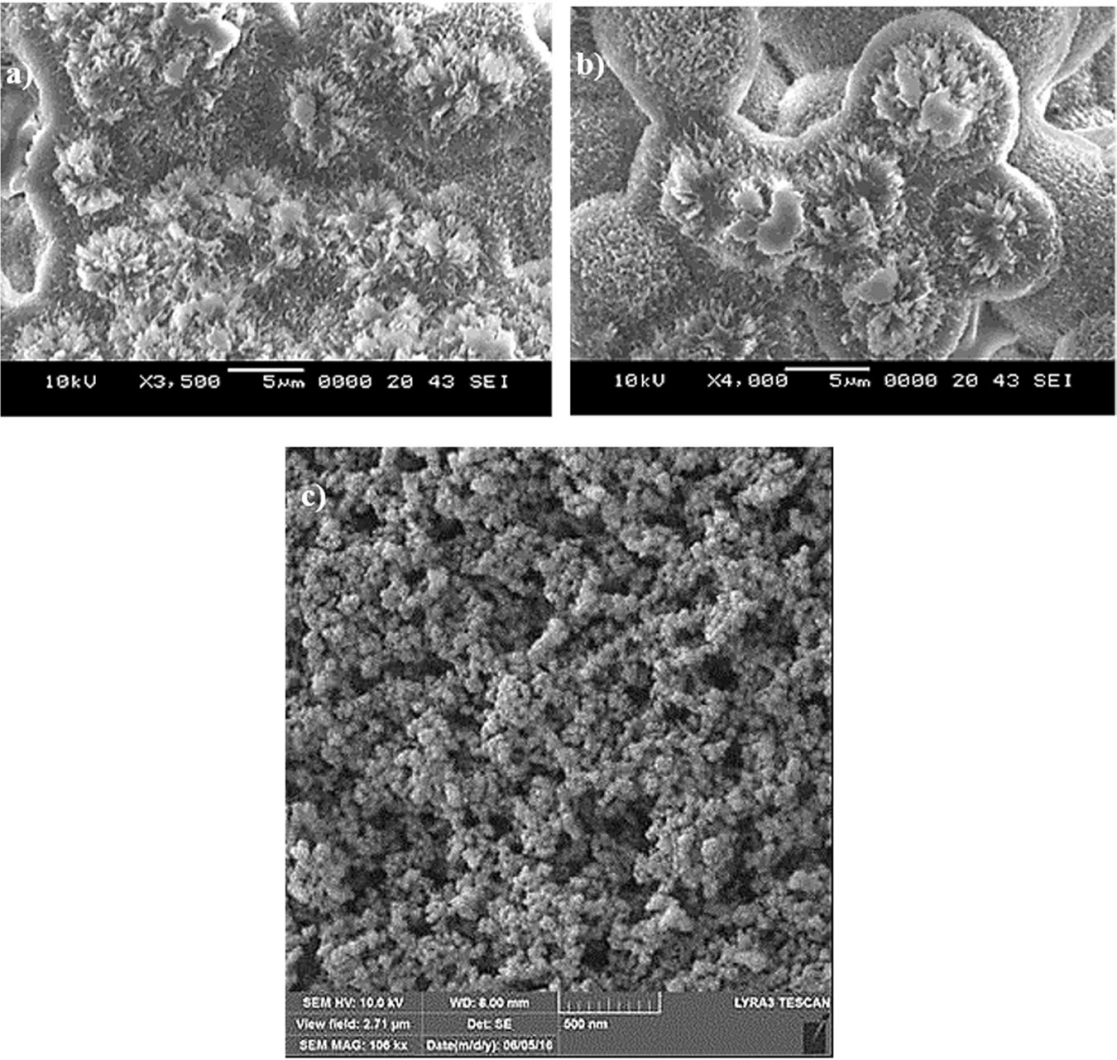
SEM micrographs of the solution-crystallized polycarbonate surface before and after functionalized particles deposition: (**a**) crystallized polycarbonate surface showing hierarchically distributed spherules and (**b**) crystallized polycarbonate surface depicting fibril-like structures formed on the spherules, and (**c**) micrograph of the surface after functionalized silica particles deposition.

**Figure 5:**
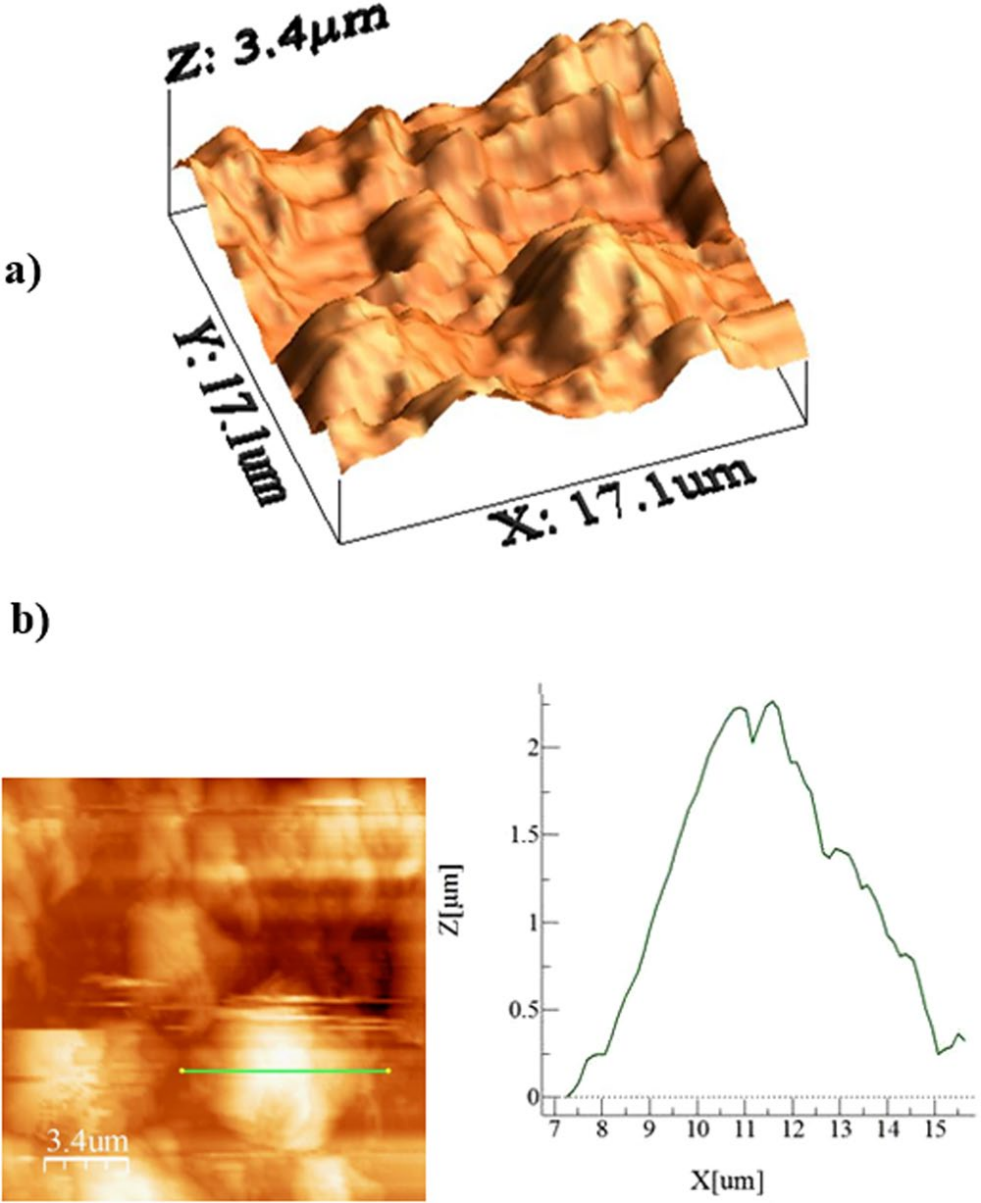
AFM image and line scan of the solution-crystallized surface and that deposited with functionalized silica particles: (**a**) 3D image of the surface and (**b**) line scan on the surface.

Figure (4) shows scanning electron microscopy (SEM) micrographs of the crystallized polycarbonate surface. A hierarchical structure with the presence of spherules (Fig. (4a)) and fine-size fibrils (Fig. (4b)) created texture on the crystallized surface. Figure (4c) shows SEM micrographs of the surfaces after deposition of the functionalized silica nanoparticles. Deposited surface composed of closely spaced some porous like textures. Because TEOS was used to synthesize the silica particles, the functionalized shell slightly alters the surface roughness of the particles^30^, as the condensing monomer units grew at a faster rate than the nucleation rate^44^; hence, the rate of formation of new nuclei was reduced. This resulted in aggregation and adhesion of the particles. However, the hydroxyl groups on the surface of the functionalized silica particles had different moieties and different reactivity towards the modifier molecules. The modified silane caused side reactions and condensation on the silica surface^49^, which contributed to the agglomeration of the functionalized particles. The surface texture characteristics are shown in the AFM images in Fig. (5). The surface is composed of spherule-like structures with fine-size texture resembling the functionalized silica particles (Fig. (5a)). The line scan of the surface reveals the presence of spheroid-like structures and nanosize silica particles (Fig. (5b)). Peaks corresponding to the nanosize silica particles appear in the line scan (Fig. (5b), indicating the spherule- and fibril-like texture. The average surface roughness is on the order of 3.4 μm.

**Figure 6:**
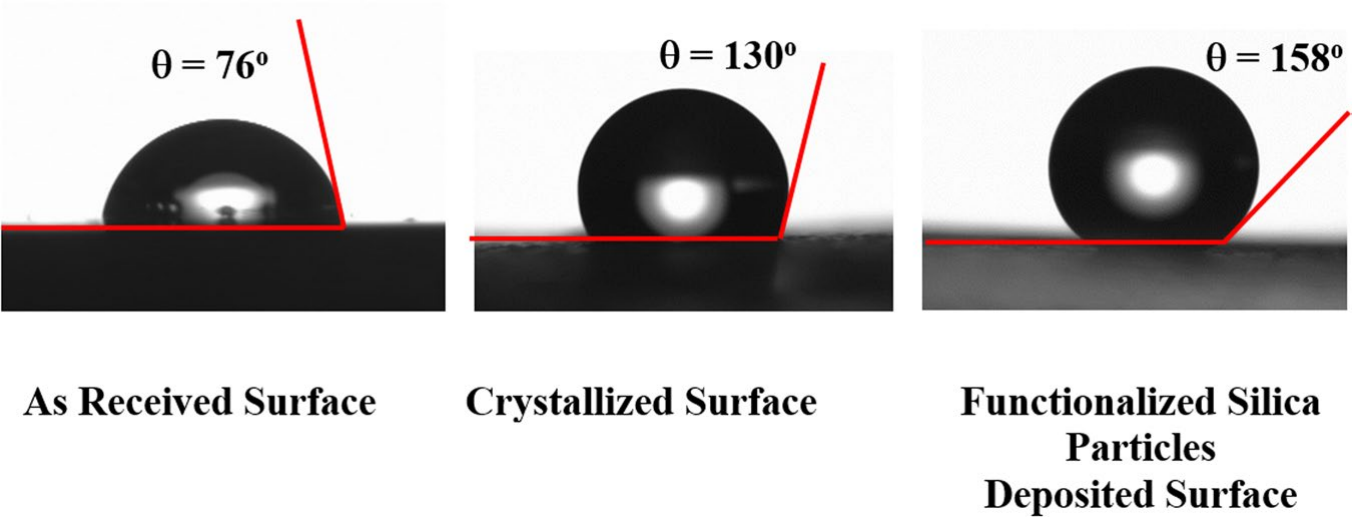
Sessile water droplet images and contact angles on the surfaces of as received polycarbonate, crystallized polycarbonate, and functionalized silica particles deposited crystallized polycarbonate samples.

The contact angle and contact angle hysteresis were measured at several locations on the surface using a goniometer, adopting the procedure introduced in a previous study^45^. The contact angle and contact angle hysteresis measured were found to be on order of less than 2%. Figure (6) shows water droplet contact angel on as received, solution crystallized, and solution crystalized and functionalized silica particles deposited surfaces. In line with a previous study^38^, dynamic contact angle measurements resulted in a water droplet contact angle on the order of 130° with contact angle hysteresis on the order of 31° for solution crystallized surface. However, dynamic contact angle remains within 158° ± 3° with contact angle hysteresis is on the order of 2°. Consequently, the functionalized silica particle deposition improves droplet contact angle and significantly reduces contact angle hysteresis of crystallized polycarbonate surface.

### Droplet Rolling on a Hydrophobic Surface

The contact angle hysteresis and inclination angle of the hydrophobic surface influences the contact line dynamics in terms of droplet attachment, rolling and sliding. In this case, a droplet with high contact angle hysteresis gives rise to a high retention force, and the droplet attaches on the hydrophobic surface despite the high inclination angle of the surface^50^. In addition, the shear force developed at the interface of the droplet and the hydrophobic surface contributes to droplet pinning on the surface during inclination of the surface. However, when the retention, due to droplet adhesion, and frictional forces become less than the gravitational force, the droplet can roll off and/or slide on the inclined hydrophobic surface, which is the case for low contact angle hysteresis. Droplet movement on the surface is influenced by the droplet bulging/puddling under gravitational force^51^. The droplet size is critical for determining droplet bulging and geometric changes during movement on the inclined surface. In this case, a droplet with a diameter less than the capillarity length (${\kappa }^{-1}=\sqrt{\frac{\sigma }{\rho g}}$, where ${\kappa }^{-1}$ is the capillarity length, *σ* is the surface tension, *ρ* is the density, and *g* is the acceleration due to gravity) remains spherical as the droplet rolls/slides on the inclined surface. On the other hand, a droplet with a diameter larger than the capillarity length can also roll and slide; however, the location of the droplet center of mass changes during rolling on the hydrophobic surface due to the retention force at the droplet-solid surface interface. Hence, the advancing and receding angles of the droplet vary during rolling for a fixed droplet volume. The change in the location of the center of mass results in droplet wobbling, i.e., the droplet height temporarily changes during rolling. Droplet wobbling influences the internal fluidity of the droplet, which alters the rotational characteristics of the droplet. Therefore, a droplet with a diameter larger than the capillarity length experiences bulging, eventually forming a puddle, which may result from the balance between the capillarity and gravitational forces. The droplet puddle thickness (*h*) was formulated previously^52^ as $\sqrt{2(1-\,\cos \,\theta )\frac{\sigma }{\rho g}}$, where *θ* is the droplet contact angle. In addition, the droplet on an inclined hydrophobic surface undergoes elastic deformation, and the resulting net force defines the droplet acceleration. The details of force balance and formulation of droplet rotational speed are provided in the supplement (S1).

**Figure 7:**
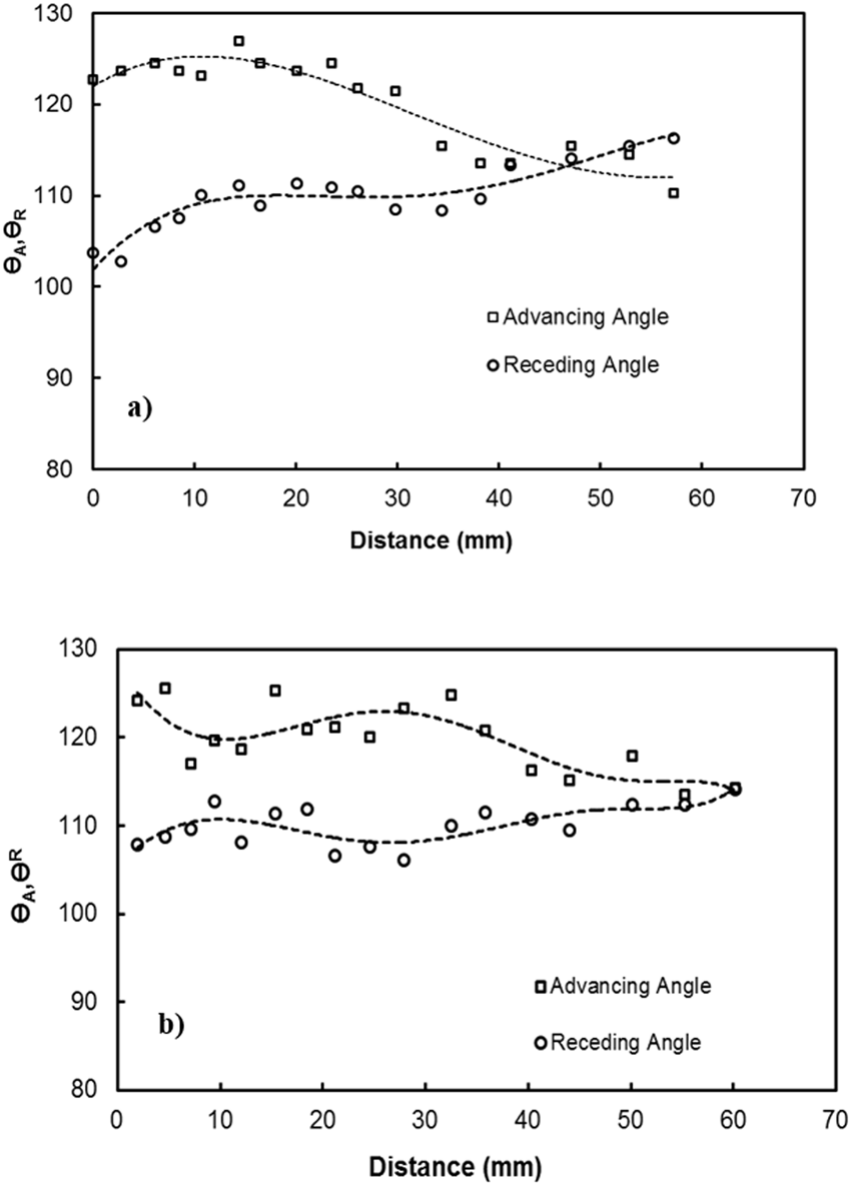
Advancing and receding angles of the water droplet on the hydrophobic surface: (**a**) clean surface and (**b**) dusty surface.

**Figure 8:**
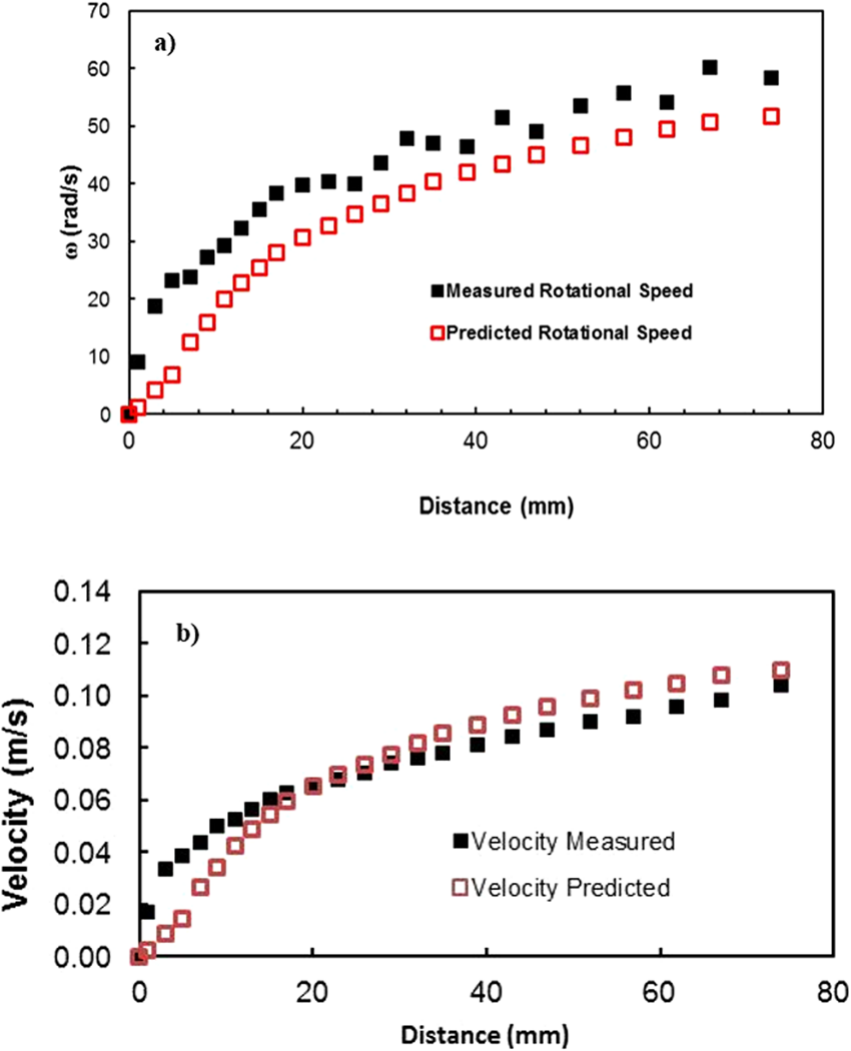
Rotational speed (**a**) and translational velocity (**b**) of a water droplet predicted analytically (S1 and S2) and obtained from a high-speed camera for the clean hydrophobic surface. Droplet volume is 40 μL, and inclination angle is 3°.

The prediction of the rotational speed of the droplet obtained analytically (S1) and that measured using a high-speed camera were compared. Note that the droplet volume is 40 μL, the inclination angle of the surface is 3°, and the variation in the dynamic contact hysteresis with distance along the inclined surface is incorporated in the calculations (S1). The advancing and receding angles of the droplet along the inclined surface are shown in Fig. (7a and b) for clean and dusty hydrophobic surfaces. Figure (8a) shows the rotational speed along the distance on the clean hydrophobic surface. The rotational speed (ω) of the clean hydrophobic surface predicted analytically (S1) agrees well with the experimental data obtained from the high-speed camera. Note that the experiments related to high-speed camera records were repeated five times, and the error related to the rotational speed obtained from the high-speed camera data is on the order of 4%.

On the other hand, when the droplet rolls off, it suffers from the energy dissipation due to fluid friction, body deformation during wobbling, retention force from adhesion of the droplet, and frictional contact between the droplet and the hydrophobic surface. Although the shear due to the rate of fluid strain developed across the contact surface between the water droplet and the hydrophobic surface is small^53^, it contributes to energy dissipation during droplet rolling. The details of energy balance and formulation of the droplet translational velocity are provided in the supplement (S2).

**Figure 9:**
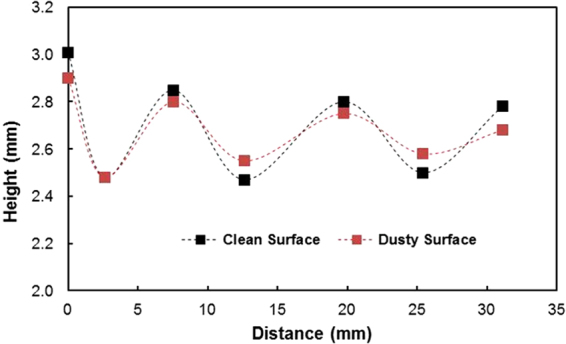
Droplet height along the clean and dusty hydrophobic surface.

**Figure 10:**
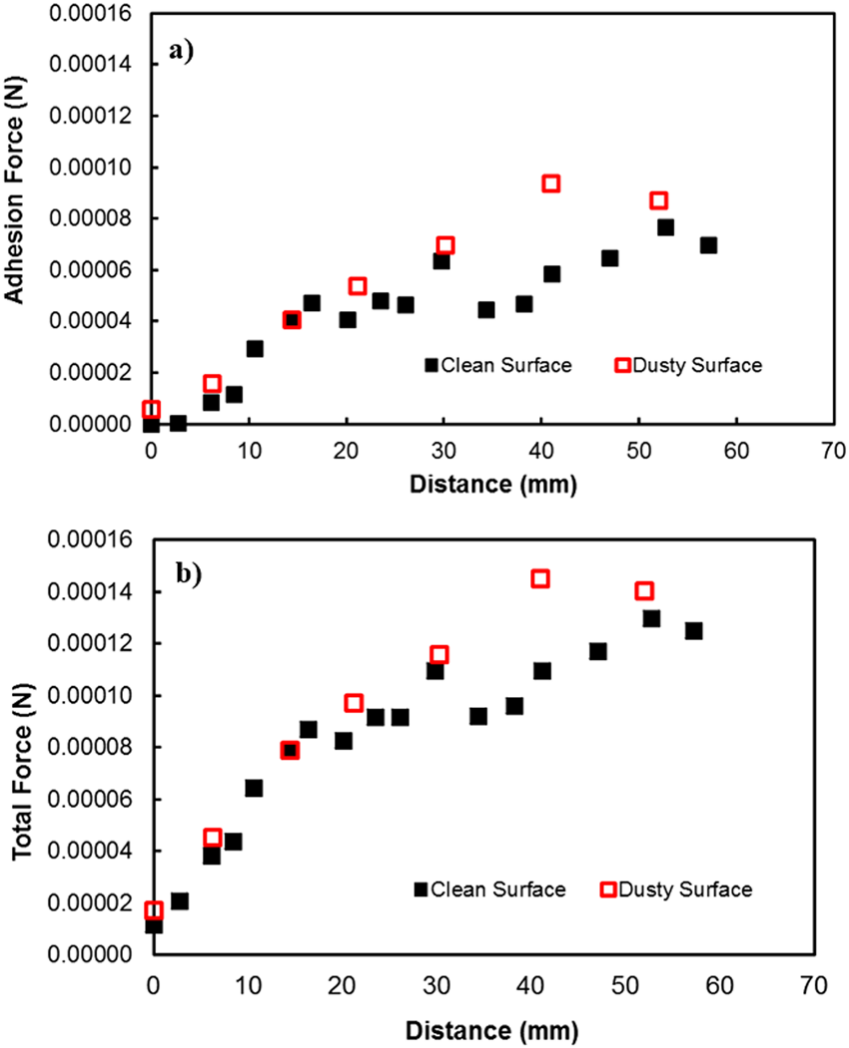
Forces acting on the hydrophobic surface during droplet motion on clean and dusty surfaces: (**a**) retention force and (**b**) total retention force. The forces were calculated analytically (S1) after determining the droplet advancing and receding angles and the droplet diameter at each location on the hydrophobic surface.

**Figure 11:**
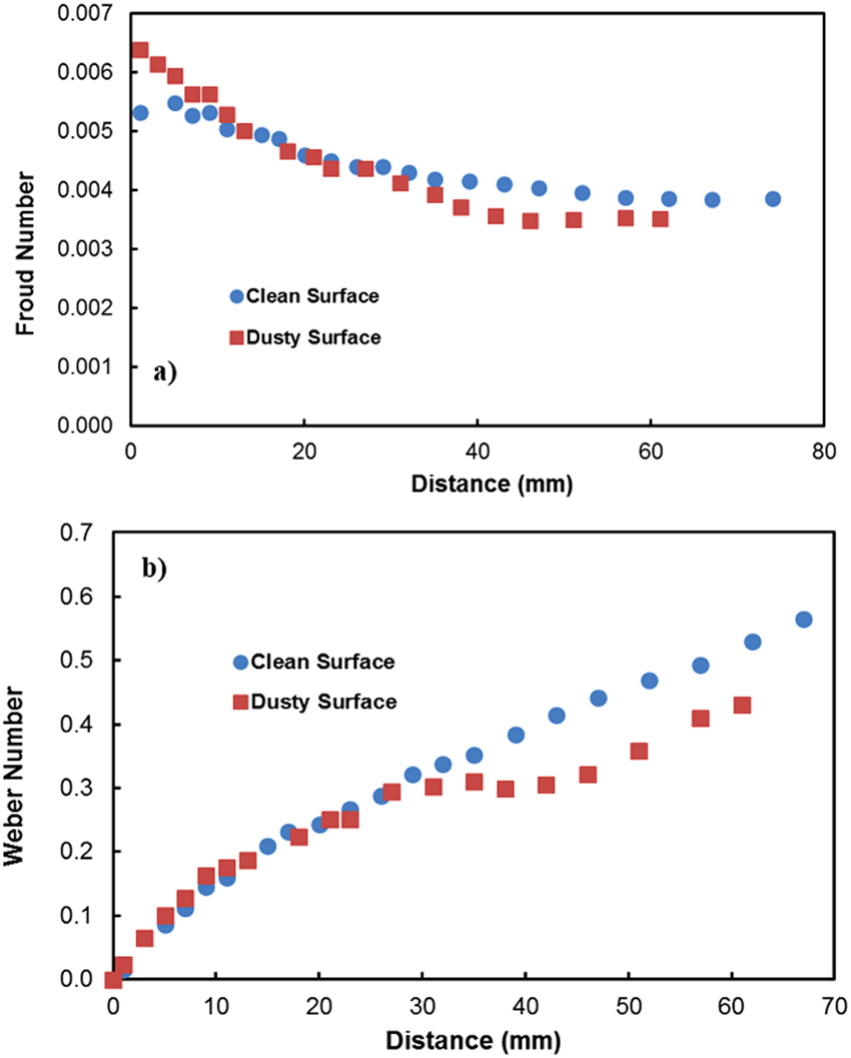
Variation of Froude and Webber numbers along distance: (**a**) Froude Number variation. (**b**) Webber Number variation.

Figure (8b) shows the droplet translational velocities relative to the hydrophobic surface for the clean hydrophobic surface predicted from the analytical formulation (S2) and obtained from experiment incorporating the high-speed camera data. The droplet velocity increases sharply with the distance along the inclined surface, and the increase in velocity reduces as the distance increases for the hydrophobic surface. This is attributed to one or all of the following: (i) the increasing droplet velocity increases the energy dissipated due to air drag, (ii) energy is dissipated due to droplet wobbling on the hydrophobic surface, and (iii) air drag and droplet wobbling alter the advancing and receding angles of the droplet at the surface, which in turn increases the energy dissipation due to droplet retention on the surface. However, energy dissipation from droplet wobbling is expected to reduce due to differences between the maximum heights of the droplet, which becomes small with increasing distance along the clean hydrophobic surface (Fig. (9)). Consequently, droplet wobbling decreases as the distance along the inclined hydrophobic surface increases. In addition, the droplet translational velocity predicted analytically (S2) and that obtained from experiment are in good agreement, provided that some small discrepancies occur between both results. These discrepancies are mainly attributed to energy dissipation from the large wobbling of the droplet with distance along the clean hydrophobic surface. Therefore, large wobbling of the droplet after rolling initially gives rise to high energy dissipation due to the work done by the deformation of the droplet body. On the other hand, the geometric features of the droplet change under the influence of gravity and surface tension force. The exact shape of the droplet depends on the balance between the gravitational force, which favors increasing contact area, and capillarity force, which opposes droplet bulging and reduces the contact area between the droplet and underlying surface. During droplet wobbling, the gravitational force lowers the droplet center of mass by a distance *λ*, and the difference in energy from a perfect sphere, which is tangent to the plane of the solid surface, can be approximated by *σλ*^2^ ≅ *ρgR*^3^,^23^, where *R* is the droplet radius and *γ*_*f*_ is the surface tension of the droplet liquid. The contact length (*l*) between the droplet and the solid surface due to droplet wobbling is related to $\,l=\sqrt{R\lambda }$. The minimization of the energy difference was estimated using the contact length, which results from $\,\rho g\lambda \sim {\gamma }_{f}{l}^{2}/{R}^{3}$. This led to the contact length in the form of $\,l\cong {R}^{2}/\sqrt{\frac{\gamma f}{\rho g}}$, which is similar to the estimation reported in a previous study^23^. The term $\sqrt{\frac{{\gamma }_{f}}{\rho g}}$ represents the capillary length. After mathematical rearrangement, the shift in the droplet center of mass (*λ*) can be reduced to ∼ *R*^3^ $/\frac{{\gamma }_{f}}{\rho g}$. The droplet height changes because of wobbling during rolling (Fig. (9)). The average difference between the maximum and minimum peak height is on the order of 0.4 mm. This agrees with that obtained from the relation $\lambda \approx {R}^{3}/\frac{{\gamma }_{f}}{\rho g}$, which is on the order of 0.43 mm for a 3 mm diameter droplet. Moreover, the dynamic advancing angle increases along the surface, while the receding angle of the droplet reduces with increasing distance along the surface. Hence, the retention force (*F*_*ad*_) on the inclined surface increases with increasing distance (Fig. (10a)). Figure (10b) shows the total resistance force (${F}_{tot}={F}_{ad}+{F}_{\tau }+{F}_{f}+{D}_{a}$) determined analytically (S1) along the distance on the hydrophobic surface. The behavior of the total force is similar to that of the retention force, and the magnitude of the total force is close to that of the retention force. Consequently, the retention force dominates over the summation of the other forces, including friction, viscous, and drags forces, on the hydrophobic surface. The variation in the advancing and receding contact angles of the droplet along the inclined surface is associated with the increased acceleration of the droplet. In this case, the droplet kinetic energy dominates over the energy dissipation due to the resisting force resulting from droplet retention. This reduction lessened droplet wobbling on the inclined surface, i.e., the variation in the droplet height with distance was reduced (Fig. (10)). Because the rotational Bond number ($\frac{\rho {\omega }^{2}{R}^{3}}{8\sigma }$, where *ρ* is the water density, *R* is the droplet radius, *ω* is the angle of rotation and *σ* is the surface tension) is related to ω^2^, the difference between the maximum and minimum droplet heights remained low for large rotational Bond numbers. In the early rolling period, the droplet rotational velocity remains low on the hydrophobic surface (Fig. (10)). The low speed gives rise to the large wobbling of the droplet. The difference between the maximum and minimum peak heights of the droplet decreases as the droplet rotational speed increases along the hydrophobic surface. The ratio of the translational speed (V) to the rotational speed (ωR) influences droplet wobbling during rolling^27^; in the present study, the rotational and translational speeds are of similar orders, i.e., ωR/V ∼ 0.98. In addition, the ratio of the rotational speed over the translational speed is critical to the relative magnitude of the dynamic pressure generated between the droplet center and the ambient pressure on the droplet^54^. In this case, for the condition $\phi =\frac{{\rm{\Delta }}\rho {\omega }^{2}{R}^{2}}{{\rho }_{a}{V}^{2}}\gg 1$ (where Δρ is the density difference between the droplet liquid and droplet ambient gas and *ρ*_*a*_ is the droplet ambient gas density), the dynamic pressure associated with the translational speed does not have a significant effect on droplet wobbling, which is consistent with previous findings^54^, i.e., in the current study, *φ* is on the order of 900; therefore, the dynamic pressure does not significantly affect droplet wobbling. On the dusty hydrophobic surface, the retention force is larger than that on the clean surface. This behavior is attributed to the change in the advancing and receding angles of the droplet by the dust particles (Fig. (7b)); in which case, the dust particles modify the three-phase contact line on the hydrophobic surface. Figure (11a and b) show the Froude ($Fr=\frac{V}{\sqrt{gL}}$, *V* is the velocity, *g* is the gravitational acceleration, and *L* is the distance) and the Weber ($We=\frac{\rho L{V}^{2}}{\gamma }$, where *ρ* is the fluid density, *L* is the distance, *V* is the velocity, and *γ* is the air-liquid surface tension) numbers for clean and dusty hydrophobic surfaces. The Froude number reduces to attain almost steady value with increasing distance along the hydrophobic surface. Since the Froude number is the ratio of inertia over the gravitational force, droplet almost reaches the terminal velocity with increasing distance. Similar arguments are also true for the dusty hydrophobic surface (Fig. (11a)), provided that the Froude number remains slightly lower for the dusty surface. This is attributed to the attainment of the smaller translational velocity of the droplet on the dusty surface than that corresponding to the clean surface. In addition, the weight of the droplet, after picking the dust particles from hydrophobic surface, contributes to the reduction of the Froude number; in which case, the weight gain of the droplet via addition of the dust particles is in the order of 2%. The Weber number remains small along the distance (Fig. (11b)) and the maximum value is less than the critical value for droplet breaking on the surface^55^.

### Droplet Dynamics on a Dusty Hydrophobic Surface

**Figure 12:**
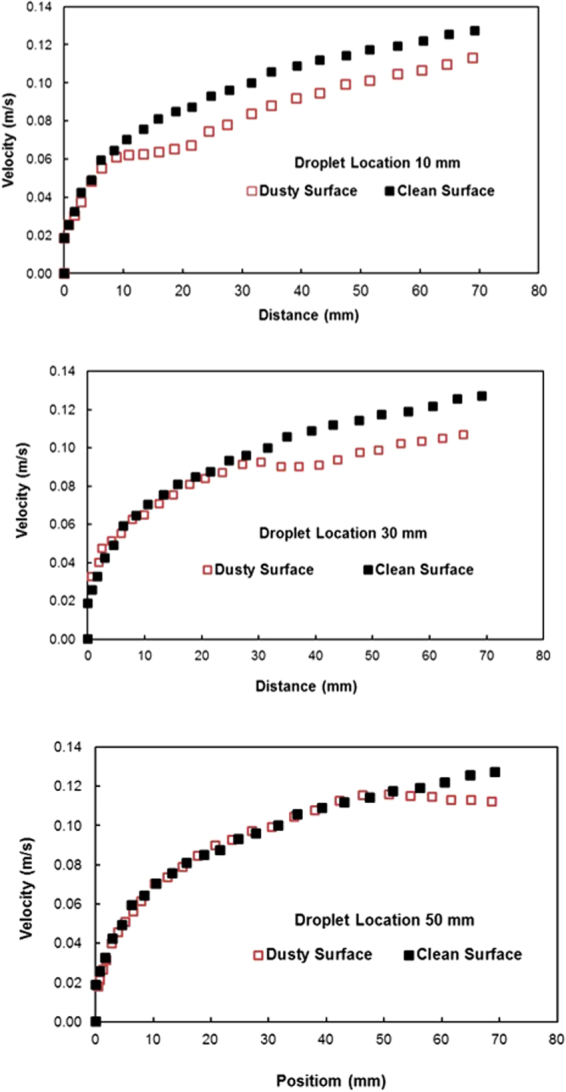
Translational velocity of the droplet on clean and dusty surfaces for various droplet locations. Droplet location is the distance between droplet initiation and the start of dusty region on the hydrophobic surface.

**Figure 13:**
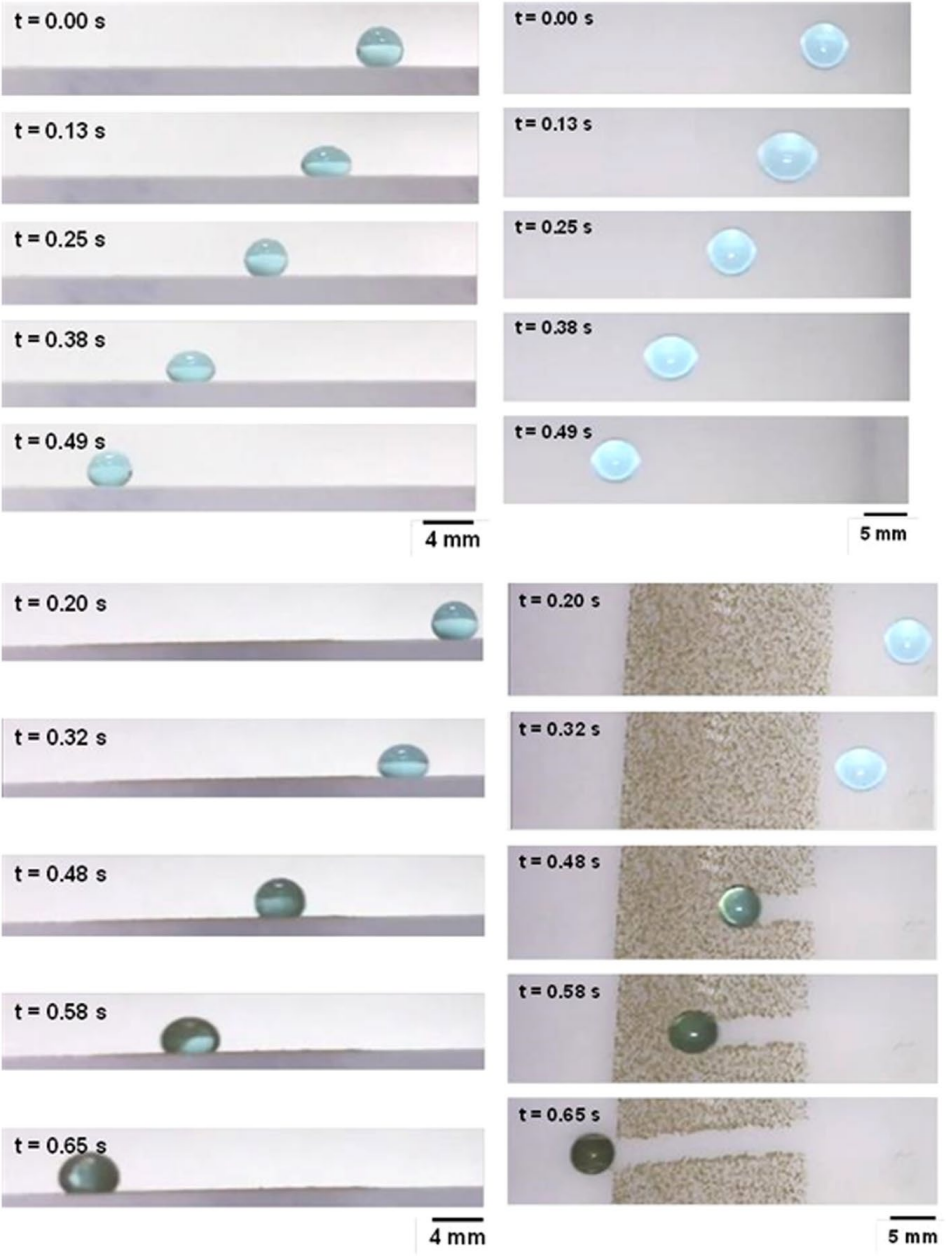
Side and top images of the droplet obtained from a high-speed camera recording for clean and hydrophobic surfaces at various durations.

**Figure 14:**
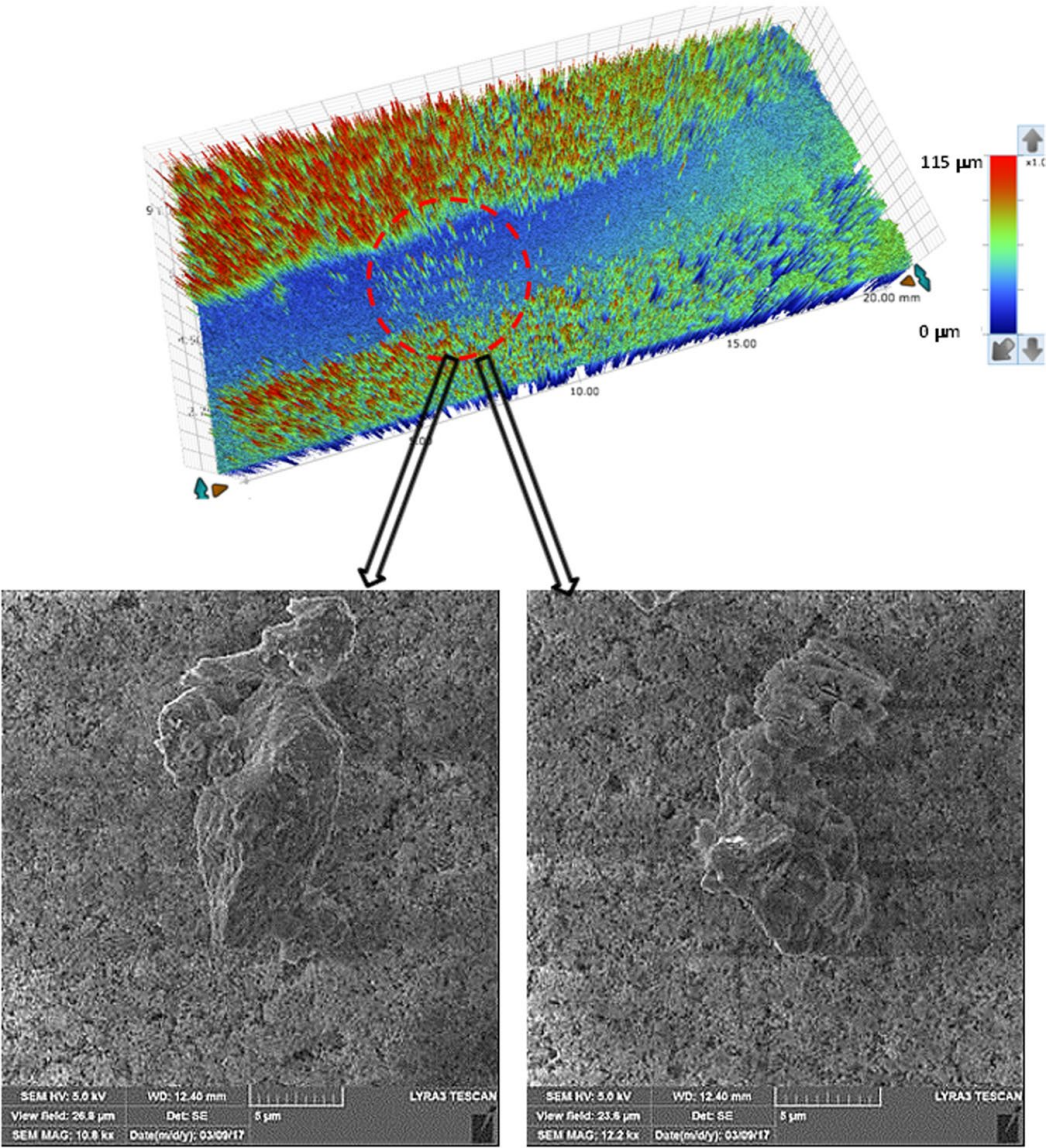
3D optical image of the droplet path and SEM micrographs of dust particle residue left on the droplet path. The red circle depicts the dust particle residue along the droplet path.

**Figure 15:**
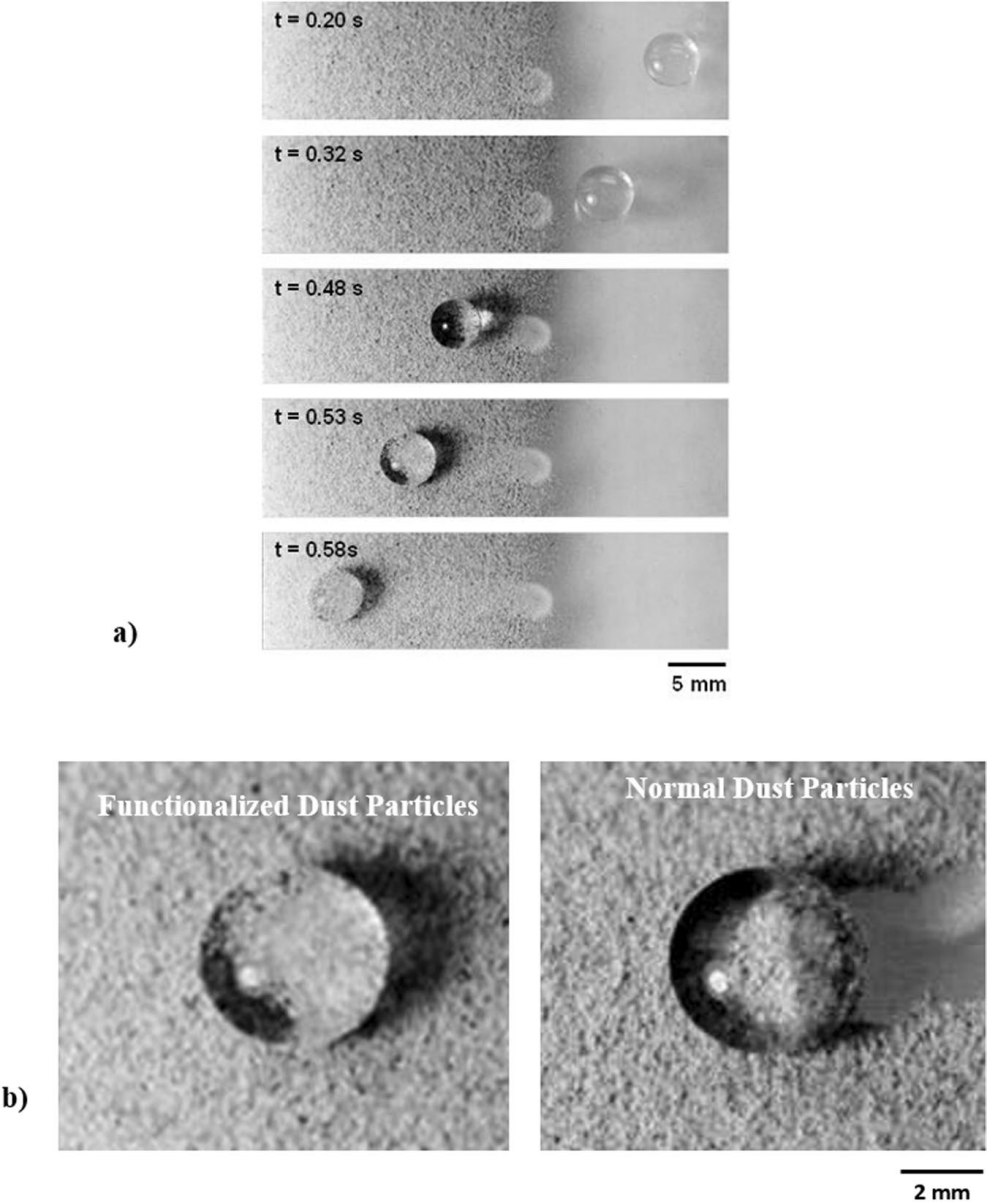
Optical images of a droplet on the hydrophobic surface with functionalized dust particles: (**a**) droplet on the hydrophobic surface with functionalized dust particles at different time duration and (**b**) top image of the droplet on the surface with functionalized and normal dust particles.

**Figure 16:**
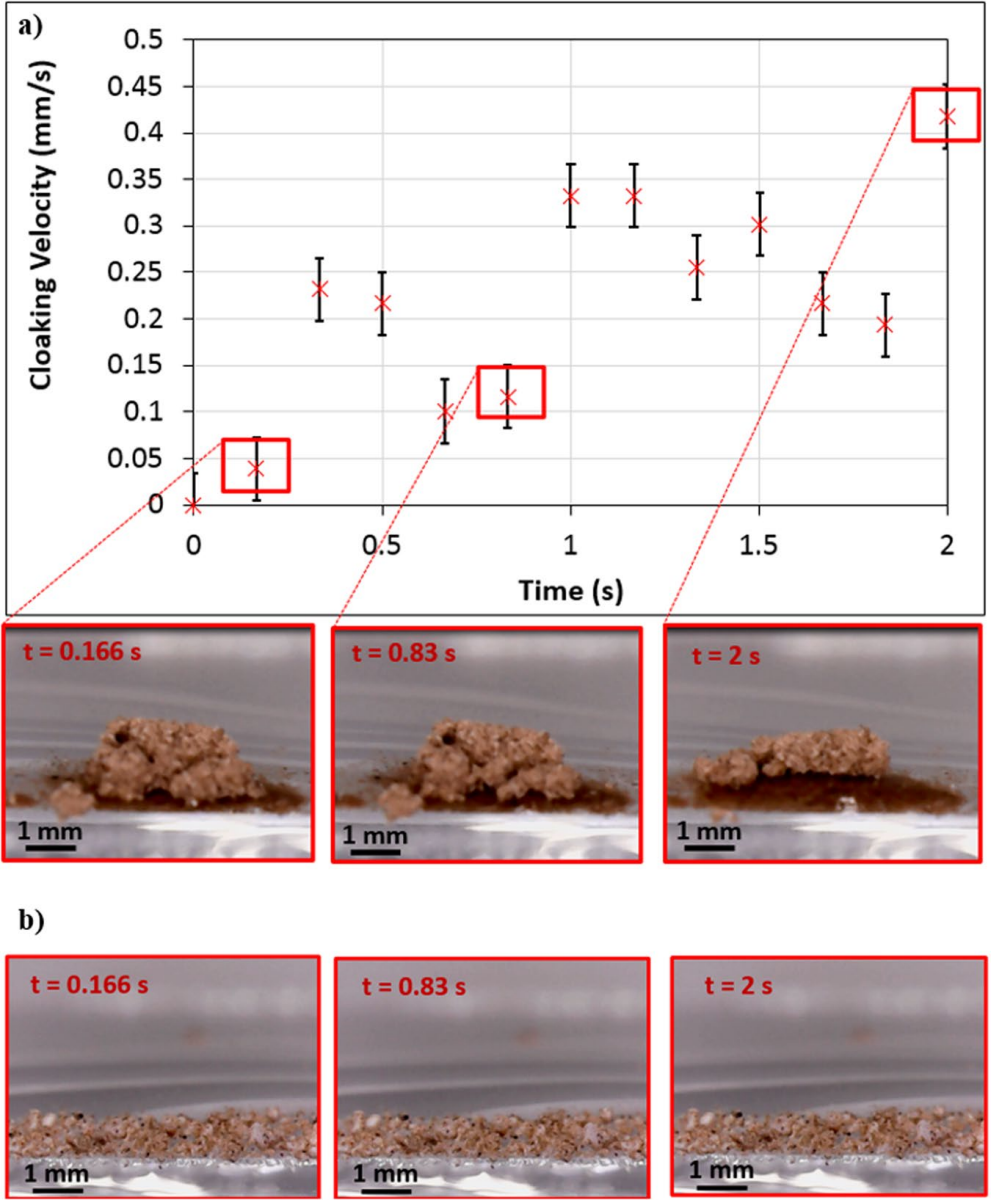
Water cloaking of normal and functionalized dust particles: (**a**) cloaking velocity and cloaking images of normal dust particles at different duration and (**b**) non-cloaking images of functionalized dust particles at different durations. Water does not cloak the functionalized dust particles for extended periods.

Figure (12) shows the droplet translational velocity along the hydrophobic surface in the presence of dust particles for three cases incorporating different length scales for droplet acceleration. It should be noted that the location at which droplet rolling is initiated is changed during the experiments. This arrangement provides varying localized acceleration of the droplet prior to reaching the dust particles on the inclined hydrophobic surface. The droplet translational velocity sharply increases towards the location of the dust on the surface. Once the droplet reaches the dust region, the droplet translational velocity increases gradually over the length of the dusty region. The droplet translational velocity increases sharply upon leaving the dusty region. Therefore, droplet acceleration is suppressed by the dust particles on the surface. This may be attributed to one or all of the following: (i) the adhesion of the dust particles on the hydrophobic surface, despite the fact that the adhesion for individual dust particles is small, and (ii) the presence of the dust particles increases the friction between the droplet and the surface due to the enhancement in the surface roughness by the dust particles—in which case, the dust particles act as an additional texture with a large texture height on the hydrophobic surface. Figure (13) shows high-speed camera images of the top and side views of the droplet at different intervals when the droplet is in the dusty region. The dust particles are picked up by the droplet as the droplet moves along the dusty region. The droplet picks up almost all of the dust particles along its path. To assess the dust particle residue left on the hydrophobic surface after droplet motion, a microscopic 3D image of the droplet path was taken and is shown in Fig. (14). In general, the droplet picks up almost all of the dust particles on its path; however, few dust particles remain on the hydrophobic surface along the droplet path (Fig. (14)). Further examination was carried out to determine the cause of the dust residue remaining on the droplet path. SEM micrographs of typical dust residue are also shown in Fig. (14), and Table 1 gives the elemental composition of the dust residue. The size of the typical dust residue is on the order of 2 μm, and it has a shape similar to the other dust particles. In addition, the elemental composition (Table 1) suggests that silica is the main compound in the dust particles. Therefore, the adhesion of dust residue on the hydrophobic surface is expected to be on the same order as those of the other dusts. Consequently, the possible explanation for the presence of dust residue along the droplet path is neither the shape effect nor the elemental composition of the dust particles, but the surface energy of the dust particle residue. Dust particle(s) can randomly obtain a low surface energy, which may be associated with the prolonged residence of some of the dust particles in air in the region close to the Gulf Sea. To assess the influence of the surface energy on the dust particles that are picked up from the hydrophobic surface by the water droplet, further experiments were carried out. In this case, the dust particles are functionalized by a surface coating of trichloro(1 H,1 H,2 H,2H-perfluorooctyl) (PFOTS) via chemical vapor deposition, in line with a previous study^56^. Figure (15a) shows optical images of the droplet path when functionalized dust particles are present on the hydrophobic surface. Optical images of the droplet path on the hydrophobic surface when normal dust particles are present are also shown for comparison. The amount of residue on the droplet path from the functionalized dust particles is significantly higher than that from normal dust particles. In addition, the functionalized dust particles picked up by the water droplet remain at the surface of the droplet rather than mixing with the droplet fluid (Fig. (15b)), unlike normal dust. This demonstrates that the wetting state of the dust particles is critical for the removal of the dust particles from the hydrophobic surface by the water droplet via the droplet liquid cloaking the dust particles during droplet movement on the hydrophobic surface. Therefore, an experiment was carried out to assess the water cloaking of the dust particles. The water cloaking velocity of the dust particles and wetting height were obtained from the high-speed camera data. Figure (16a) shows the stages of the water cloaking of the dust particles and the cloaking velocity. The cloaking velocity first increases rapidly and then reduces as time progresses. Because water film cloaking occurs opposite to gravity, as the weight of the water film cloaking the dust particles increases, the net driving force opposing gravity for cloaking decreases. Therefore, the water cloaking experiment was extended to the functionalized dust particles for comparison. Water does not cloak the functionalized dust particles, as seen in Fig. (16b). This behavior is associated with the spreading rate of the water film at the dust particle-air interface. The spreading coefficient (${S}_{op(a)}={\gamma }_{pa}-{\gamma }_{pw}-{\gamma }_{wa},$ where *γ*_*pa*_ is the interfacial energy at the dust particle-air interface, *γ*_*pw*_ is the interfacial energy at dust particle-water interface, and *γ*_*wa*_ is the interfacial energy at the water-air interface) must be greater than zero for water to cloak the outer surface of the dust particles. Although the interfacial energy between the dust particle and air is unknown, the condition ${\gamma }_{pa} > ({\gamma }_{pw}+{\gamma }_{wa})$ should be satisfied for water cloaking. Note that the dust particles were compacted into a pellet, and the surface energy of the dust pellet can be determined through experiments incorporating the Owens–Wendt (OW) method^57^. The surface energy of the pellet was determined to be on the order of 750 mJ/m^2^, which is between the surface energy of calcite (347 mJ/m^2^,^58^) and silica (1500 mJ/m^2^,^59^). The actual surface energy of the dust particles may slightly differ from that of the measured value; however, it should remain within a similar order of magnitude because of the major constituting elements, silica and calcite. Because ${\gamma }_{wa}=72\,mJ/{m}^{2}$, in any case, *γ*_*pw*_ should be less than 678 mJ/m^2^. On the other hand, water spreading on the dust particles occurs into two stages. In the first stage, the balance between the surface tension gradient and the shear stress at the water-dust interface results in a monolayer of water spread on the dust particle. In the second stage, the location of water spreading follows Joos’ law^60^, and the spreading velocity can be related to ${V}_{s}\propto {(3{S}_{ow(a)}/4\sqrt{{\mu }_{o}{\rho }_{o}})}^{1/2}{t}^{-1/4}$), where μ_o_ is the dynamic viscosity of water, ρ_o_ is the density of water, and S_ow(a)_ is the spreading coefficient of water on the dust particles^53^. The dissipating force during water spreading around a dust particle can be approximated by the Ohnesorge number ($Oh={\mu }_{o}/\sqrt{{\rho }_{o}a{\gamma }_{oa}}$), where *a* is the characteristic size of the dust particle^53^, which can be considered to be the equivalent diameter^61^. For an average dust particle size of 1.2 μm, *Oh* well exceeds unity (*Oh* > 1), which implies a large dissipation force for water cloaking of the dust particle. The cloaking rate is associated with cloaking time in the form of ∼*k*_*m*_*t*^*1/4*^, where *k*_*m*_ is the cloaking factor^61^, and cloaking is not possible if *k*_*m*_*t*^*1/4*^ < 1. In the present case, *k*_*m*_*t*^*1/4*^ was determined to be greater than unity for normal dust particles. Moreover, the cloaking velocity was determined from high-speed camera data, which is shown Fig. (15a), and its average value is on the order of 0.3 × 10^−3^ m/s. From the average cloaking velocity, the duration of complete cloaking of the dust particle within the range of 1.2–10 μm is on the order of 0.0315 s. Note that the cloaking velocity is inversely related and the cloaking time; in which case, the relation for the cloaking velocity can be approximated in the form of ∼*Ct*^*−0.5*^, where *C* is a constant that varies with the shape of the dust and *t* is the cloaking time. Moreover, the distance corresponding to the cloaking time and travelled by the droplet on the hydrophobic surface is on the order of 9 μm, which is much less than the contact length of the 40 μL liquid droplet on the solid surface (*l* ≅ 0.002 m). Therefore, the dust particles are picked up from the hydrophobic surface by the water droplet cloaking the dust particles. The dust particles picked up by the droplet remain in the droplet fluid and mix with the droplet liquid (Fig. (15b)). On the other hand, the functionalized dust particles do not penetrate into the droplet liquid and instead remain at the droplet surface (Fig. (15b)). Consequently, the droplet fluid and the functionalized dust particles do not mix. The adhesion of the functionalized dust particles on the water droplet surface can be attributed to the electrostatic attraction developed within the deposited surface of the functionalized dust particles^62–64^.

**Figure 17:**
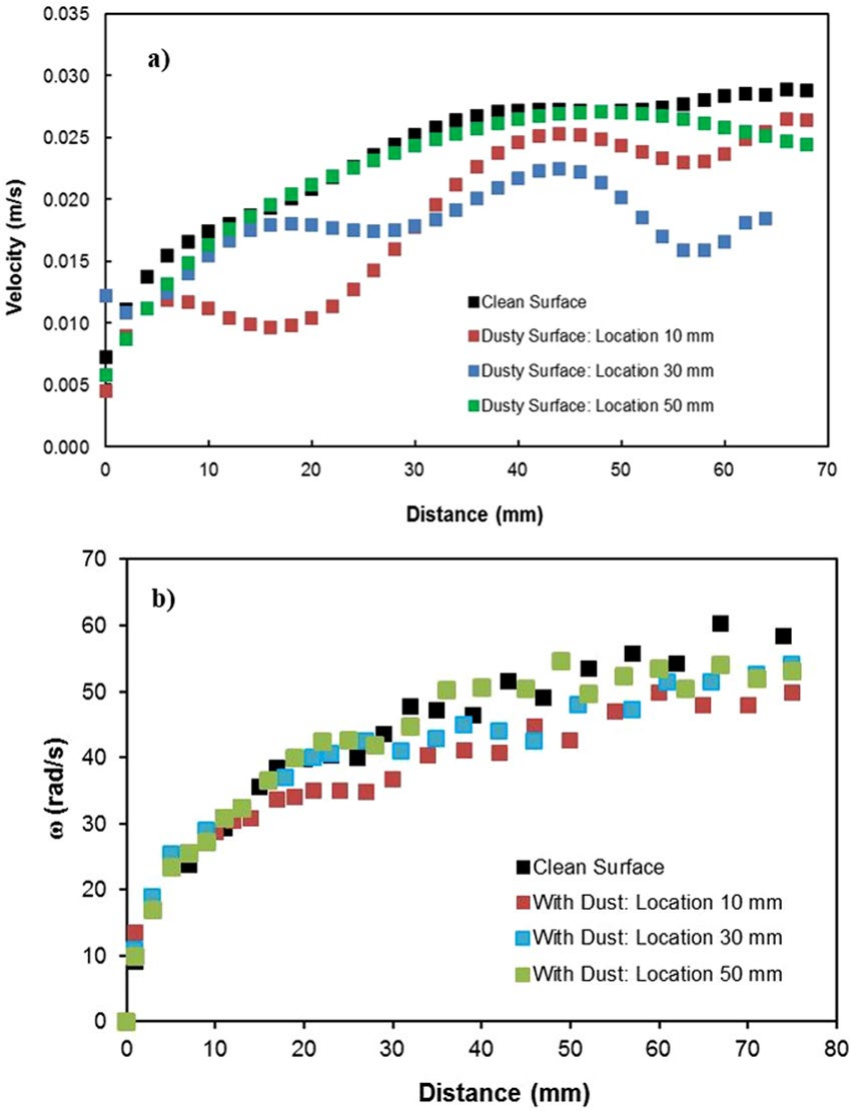
Slip velocity (**a**) and rotational speed (**b**) of the droplet on clean and dusty hydrophobic surfaces for different droplet locations. The droplet location represents the distance between the droplet initiation and the start of the dusty region.

The translational velocity of the droplet is composed of the rolling and slip velocities of the droplet. The slip velocities during droplet movement on the hydrophobic surface with and without dust particles are shown in Fig. (17a). Note that the data presented in Fig. (15a) were obtained from experiments incorporating the high-speed camera. In addition, a comparison of the rotational speed of the droplet on the hydrophobic surface with and without dust particles is shown in Fig. (17b). The rolling velocity (angular speed) reduces along the region at which the dust particles are located. This behavior is similar to that of the translational velocity (Fig. (12)), which is associated with an increased retention force between the droplet and the dusty surface (Fig. (10)). The sliding velocity of the droplet is small, on the order of 0.02 m/s, which is smaller than the translational velocity. As the droplet progresses along the hydrophobic surface, the sliding velocity slightly increases. The attainment of low sliding velocity is associated with the retention of the droplet on the hydrophobic surface, which is larger for the dusty surface than the clean surface (Fig. (10)). For the dusty surface, the retention force, resulting from the difference between the advancing and receding angles, is on the order of 80 μN, which is slightly higher than that on the clean surface (50 μN).

**Figure 18:**
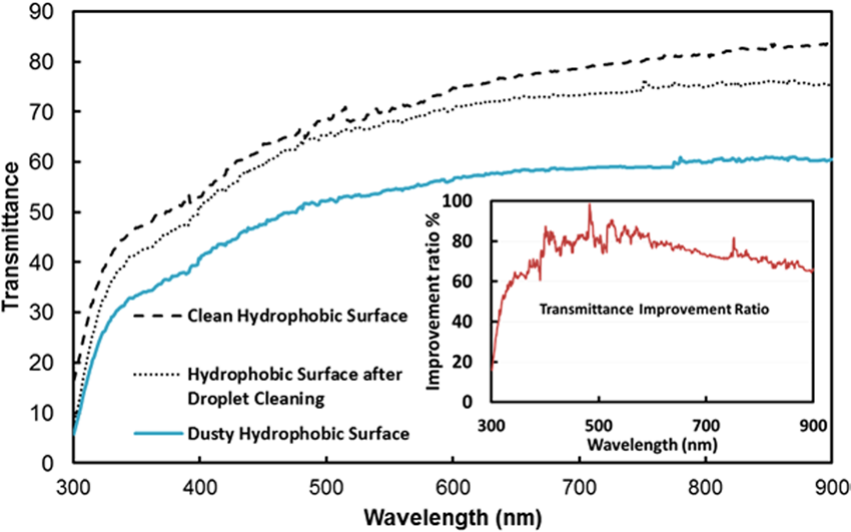
UV-visible transmittance of the hydrophobic surface before and after dust removal by water droplets. The inset figure depicts the improvement ratio of the transmittance. The ratio was determined from the difference between the optical transmittance before and after dust removal by water droplets over the difference between the optical transmittance of the hydrophobic surface before dust deposition and after removal of dust from surface by water droplets.

Figure (18) shows the optical transmittance of the hydrophobic, dusty, and self-cleaned by the water droplet surfaces. In addition, a graph of the relative ratio of the optical transmittance of the surfaces before and after dust removal from the hydrophobic surface by the water droplet is inserted in Fig. (18). The ratio is determined from the difference between the optical transmittance of the water droplet cleaned hydrophobic surface and transmittance of the dusty hydrophobic surface over the difference between transmittance of the hydrophobic surface and transmittance of the dusty hydrophobic surface ($=\frac{({T}_{Cleaned}-{T}_{Dusty})}{({T}_{Hydrophobic}-{T}_{Dusty}}$, where *T*_*Cleaned*_ is the transmittance after dust removed by water droplet, *T*_*Dusty*_ is the transmittance of dusty surface, and *T*_*Hydrophobic*_ is the transmittance of the hydrophobic surface). Dust accumulation on the surface reduces the optical transmittance of the hydrophobic surface significantly. Water droplet cleaned surface improves the optical transmittance considerably. The transmittance improvement ratio (inset graph) shows the transmittance improvement almost over 60% in average over the wavelengths of the optical radiation incorporated. Consequently, the transmittance difference between the dusty and hydrophobic surface significantly increases when cleaned by water droplet. This improvement in transmittance demonstrates the feasibility of the self-cleaning of surfaces by water droplets, which can provide a possible cleaning process for solar energy harvesting applications.

## Conclusion

The dynamics of a water droplet on an inclined hydrophobic surface were considered, and the removal of environmental dust particles from the hydrophobic surface by water droplets was examined. The dust particles were collected from the energy laboratory of King Fahd University of Petroleum and Minerals located in Dammam, Saudi Arabia. The solution crystallization of a polycarbonate surface was carried out to generate a hierarchical texture consisting of spheroids and fibrils. The resulting textured surface demonstrates hydrophobic characteristics with high contact angle hysteresis, which increases water droplet pinning and suppresses the dynamic motion of the droplet on the hydrophobic surface. To reduce the contact angle hysteresis, functionalized nanosize silica particles were deposited on the surface. The resulting surface displays a high droplet contact angle (158°) and low contact angle hysteresis (3°). The translational velocity of the droplet is composed of the rotational and slips velocities, and the rotational velocity dominates over the slip velocity along the hydrophobic surface, which is true for the inclined hydrophobic surfaces both with and without dust. The prediction of the rotational speed agrees well with that obtained from experiment. The initial location of the droplet on the hydrophobic surface (standoff distance) is important in terms of the droplet translational velocity; in which case, the translation velocity reaches an almost stable value as the distance along the hydrophobic surface increases. The initial location of the dust particles on the hydrophobic surface is also important in terms of the droplet translational velocity along the dusty region. In this case, the droplet velocity along the dusty region is lower at short distances (10 mm) than at large distances (50 mm). Consequently, the droplet velocity is not affected by the dust particles along its path when the droplet velocity attains high values prior to reaching the dusty region. The retention forces of the clean and dusty surfaces are almost similar; however, the retention force for the droplet on the dusty region is higher than that on the clean surface. On the other hand, droplet cloaking is responsible for the removal of dust particles from the hydrophobic surface by the water droplet; in which case, the time required for cloaking the dust particles is less than the transition time during which the droplet is wet along the length of the hydrophobic surface. Few dust residues are left on the droplet pathway after the droplet passes over the dusty region. The elemental composition of the dust residue is similar to that of the ordinary dust particles. The dust residues have low surface energy while giving rise to a negative spreading rate; thus, the water droplet could not cloak these particles during its transition along the pathway. The hydrophobic surface cleaned by the rolling water droplet significantly improved the optical transmittance of the surfaces over that of the dusty surface. The present study gives insight into the droplet dynamics and environmental dust particles on inclined hydrophobic surfaces in relation to self-cleaning applications. It also provides useful information on the fundamental understanding of the removal of environmental dust particles from hydrophobic surfaces by droplet motion.

## Electronic supplementary material

Geometry of Rolling Droplet

Droplet Translational Velocity
