# Supplementary material for: Water Droplet Dynamics on a Hydrophobic Surface in Relation to the Self-Cleaning of Environmental Dust
Source: Sci Rep. 2018 Feb 14;8:2984. doi: 10.1038/s41598-018-21370-5 (PMC5813023; doi:10.1038/s41598-018-21370-5)
Supplement: Supplementary file 1 — Geometry of Rolling Droplet [file 41598_2018_21370_MOESM1_ESM.pdf]

WATER DROPLET DYNAMICS ON A HYDROPHOBIC SURFACE IN RELATION TO THE SELF-CLEANING OF ENVIRONMENTAL DUST

Bekir Sami Yilbas[1,2], Ghassan Hassan[1,2], Abdullah Al-Sharafi[1], Haider Ali[1], Nasser Al-Aqeeli[1], Abdelsalam Al-Sarkhi[1]

[1]Department of Mechanical Engineering, King Fahd University of Petroleum and Minerals (KFUPM), Dhahran 31261, Saudi Arabia,

[2]Center of Research Excellence in Renewable Energy (CoRE-RE), King Fahd University of Petroleum and Minerals (KFUPM), Dhahran 31261, Saudi Arabia

*Corresponding author. Email: bsyilbas@kfupm.edu.sa; Phone: +966 3 860 4481

S1: Geometry of Rolling Droplet

The droplet puddling and wobbling modify the line of action of the net force inside the droplet and also alter the dynamic hysteresis of the droplet ($\theta_R - \theta_A$), where θ_R is the receding angle and θ_A is the advancing angle of the droplet during rolling, while changing the droplet retention force on the hydrophobic surface during rolling. Considering Figure (1s), in which the force diagram and the rolling droplet is shown, the force balance for a steadily rolling droplet around the center of mass yields:

$$mg\sin\delta - F_{ad} - F_{\tau} - F_{f} - D_{a} = \frac{2}{5}mR\omega^{2} \tag{1}$$

where m is the droplet mass; δ is the inclination angle of the hydrophobic surface; F_{ad}, F_{τ}, and F_f are the retention, shear, and frictional force between the surface and droplet during rolling, respectively; D_a is the air drag force; R is the droplet radius; and ω is the angle of rotation.

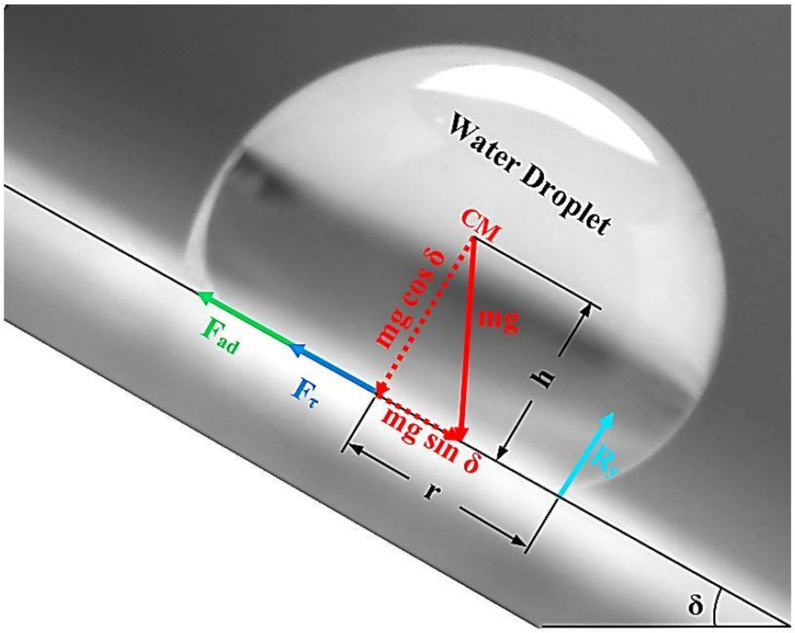

Figure 1s. High-speed camera image of a water droplet on an inclined surface and the force diagram.

The retention force was formulated previously by approximating a three-phase contact line with a single ellipse[1]. Later, a polynomial function was developed using experimental data for the dependence of the contact angle on the position along the three-phase contact line[2]. In this case, the retention force equation yields[2]:

$$F_{ad} = \frac{24}{\pi^3} \gamma_{LV} D (cos\theta_R - cos\theta_A) \quad (2)$$

where γ_{LV} is the surface tension of the liquid on the solid surface, D is the droplet diameter prior to deformation (the same area as the ellipse), θ_R is the receding angle, and θ_A is the advancing angle. Because the hydrophobic surface has texture, the roughness parameter can be introduced in Equation 2, in line with the Young-Dupre Equation[3]. Hence, Equation 2 becomes:

$$F_{ad} = \frac{24}{\pi^3} \gamma_{LV} D f (cos\theta_R - cos\theta_A) \quad (3)$$

where f is the solid surface fraction (solid-liquid contact fraction).

A shear force is generated when the droplet rolls/slides on the hydrophobic surface due to the rate of fluid strain developed along the contact surface between the water droplet and the hydrophobic surface. Therefore, the shear stress can be expressed as:

$$F_\tau = A_w (\mu \frac{dV}{dy}) \quad (4)$$

where A_w is the contact area ($A_w = \pi r^2$, where r is the contact area radius), μ is the droplet fluid viscosity, V is the flow velocity, and y is the distance normal to the contact surface. The frictional force associated with the droplet and the hydrophobic surface can be presented in terms of the normal force and the friction coefficient of the hydrophobic surface:

$$F_f = \mu_f F_n \tag{5}$$

where μ_f is the friction coefficient of the hydrophobic surface and F_n is the normal force, which is of the same order of the droplet weight (*mg,* where m is the droplet mass). To obtain the friction coefficient of the hydrophobic surface, AFM friction measurements were utilized and the average friction coefficient was determined to be 0.03.

The drag force due to air resistance as a droplet rolls on a surface is related to the pressure drag and frictional drag. However, the simplified form of the drag force for a spherical body due to air resistance is a function of the flow Reynolds number and shape factor. However, the drag force can be related to $D \cong 1/2 C_d \rho_a A_c U_T^2$, where C_d is the drag coefficient[4] and U_T is the air velocity opposing the droplet during droplet movement on the hydrophobic surface. However, it can be considered to be on the same order as the translation velocity of the droplet; in which case, $U_T \cong V$, where V is the translational velocity of the droplet. The droplet rotational speed can be obtained by rearranging Equation 1, which yields

$$\omega = \sqrt{\left(\frac{\frac{5}{2mR}\left(mg\sin\delta - \frac{24}{\pi^3}\sigma f\left(\cos\theta_R - \cos\theta_A\right) - \mu_t A_w \frac{\partial u}{\partial y} - \mu_f mg \right)}{1 + \frac{5}{4m} C_d \rho_a A_c R} \right)} \tag{6}$$

REFERENCES

1. ElSherbini, A.I. & Jacobi, A.M. Retention forces and contact angles for critical liquid drops on non-horizontal surfaces, J. Colloid and Interface Science 209, 841-849 (2006).

2.. Pilat, D.T Papadopoulos, P. Schaffel, D. Vollmer, D. Berger, R. Butt, H.J. & Dynamic measurement of the force required to move a liquid drop on a solid surface, Langmuir 28, 16812-16820 (2012).

3. Ayyad, A. H. Thermodynamic derivation of the Young–Dupré form equations for the case of two immiscible liquid drops resting on a solid substrate, Journal of colloid and interface science 346, 483-485 (2010).
4. Mccormick, B.W. Aerodynamics, aeronautics, and flight mechanics: Wiley New York (1995).
