# Supplementary material for: Water Droplet Dynamics on a Hydrophobic Surface in Relation to the Self-Cleaning of Environmental Dust
Source: Sci Rep. 2018 Feb 14;8:2984. doi: 10.1038/s41598-018-21370-5 (PMC5813023; doi:10.1038/s41598-018-21370-5)
Supplement: Supplementary file 2 — Droplet Translational Velocity [file 41598_2018_21370_MOESM2_ESM.pdf]

WATER DROPLET DYNAMICS ON A HYDROPHOBIC SURFACE IN RELATION TO THE SELF-CLEANING OF ENVIRONMENTAL DUST

Bekir Sami Yilbas[1,2], Ghassan Hassan[1,2], Abdullah Al-Sharafi[1], Haider Ali[1], Nasser Al-Aqeeli[1], Abdelsalam Al-Sarkhi[1]

[1]Department of Mechanical Engineering, King Fahd University of Petroleum and Minerals (KFUPM), Dhahran 31261, Saudi Arabia,

[2]Center of Research Excellence in Renewable Energy (CoRE-RE), King Fahd University of Petroleum and Minerals (KFUPM), Dhahran 31261, Saudi Arabia

*Corresponding author. Email: bsyilbas@kfupm.edu.sa; Phone: +966 3 860 4481

S2: Droplet Translational Velocity

Considering the conservation of energy, an equation for the droplet velocity on the hydrophobic surface can be formulated. In this case, the change in the potential energy of the droplet prior to rolling remains the same as the summation of the change in the dissipated energy and the kinetic energy of the droplet regardless of location on the inclined surface, i.e., ΔE_{Tot} - ΔE_{loss} = $\Delta E_{kinetic}$, where ΔE_{tot} ($\Delta E_{tot} = mg\Delta h$, where m is the mass of the droplet, g is the gravitational acceleration, and Δh is the elevation between the droplet location and the reference level) represents the potential energy change of the droplet along the inclined hydrophobic surface. ΔE_{loss} is the dissipation energy, which can be written in the form of $\Delta E_{loss} = \Delta E_{friction} + \Delta E_{deformation} + \Delta E_{retention} + \Delta E_{shear} + \Delta E_{air\text{-}drag}$, where $\Delta E_{friction}$, $\Delta E_{deformation}$, $\Delta E_{retention}$, and $\Delta E_{air\text{-}drag}$ are the dissipation due to i) friction (frictional energy dissipation between the droplet and surface during rolling), ii) elastic deformation of the droplet during wobbling, iv) work done against droplet retention due to dynamic contact angle hysteresis, and v) air drag during rolling, respectively. Here, the energy dissipation due to friction is $\Delta E_{friction} = \mu \Delta L F_n$, where μ is the dynamic friction coefficient between the water droplet and the hydrophobic surface, ΔL is the distance along the inclined hydrophobic surface, and F_n is the normal force due to the droplet weight. The energy dissipated during deformation of the droplet due to wobbling can be described as $\Delta E_{deformation} \sim \forall_p \gamma_L (\frac{D_{h_1} - D_{h_2}}{D_{h_1} D_{h_2}})$, where $\forall p$ is the droplet volume, γ_L is the surface tension of the droplet fluid, D_{h_1} is the instant hydraulic diameter of the droplet at a location on the inclined hydrophobic surface, and D_{h_1} and D_{h_2} are the changes in the hydraulic diameter of the water droplet along the distance ΔL on the inclined hydrophobic surface due to wobbling. The energy dissipation due to the retention force can be described as $\Delta E_{adhesion} \sim \frac{24}{\pi^3} \gamma_L D f \Delta L (cos\theta_R - cos\theta_A)$, where θ_A is the dynamic advancing angle and θ_R is the dynamic receding angle, which change along the distance ΔL during rolling of the droplet. The energy dissipation due to fluid friction because of the rate of fluid strain can be described as $\Delta E_{shear} \sim A_w (\mu_t \frac{dV_f}{dy}) \Delta L$, where A_w is the contact area ($A_w = \pi r^2$, where r is the contact area radius), μ_t is the droplet fluid viscosity, V_f is the flow velocity of the droplet, and y is the distance normal to the contact surface. It is assumed that the

maximum fluid velocity in the droplet is of the same order as the tangential velocity of the droplet. In the case of air drag loss, $\Delta E_{air-drag} = \frac{1}{2} K_L m U_T^2$, where K_L is the loss coefficient due to air drag. The tangential velocity (U_T) is determined from the angular rotation of the droplet (S1) after introducing the hydraulic radius ($D_H/2$ is the instant hydraulic diameter). The energy balance of the droplet (ΔE_{Tot} - ΔE_{loss} = $\Delta E_{kinetic}$,) allows formulation of the droplet velocity on the inclined hydrophobic surface:

$$V = \sqrt{\begin{array}{c} 2g[\Delta L sin\alpha - \mu_f \Delta L - \frac{1}{mg}\frac{24}{\pi^3}\gamma_L D f \Delta L(cos\theta_R - cos\theta_A) \\ -\frac{4\gamma_L}{\rho g \Delta L}\left(\frac{D_{h_1} - D_{h_2}}{D_{h_1} D_{h_2}}\right) - \frac{1}{mg} A_w \left(\mu_t \frac{dV_f}{dy}\right)\Delta L - \frac{1}{2g} K_L U_T^2] \end{array}} \qquad (1)$$
